# Functional reconstruction of injured corpus cavernosa using 3D-printed hydrogel scaffolds seeded with HIF-1α-expressing stem cells

**DOI:** 10.1038/s41467-020-16192-x

**Published:** 2020-06-01

**Authors:** Geng An, Feixiang Guo, Xuemin Liu, Zhifang Wang, Ye Zhu, Yong Fan, Chengkai Xuan, Yan Li, Hongkai Wu, Xuetao Shi, Chuanbin Mao

**Affiliations:** 1 Department of Reproductive Medicine Center, Third Affiliated Hospital of Guangzhou Medical University, Guangzhou, 510150 P. R. China; 2 National Engineering Research Centre for Tissue Restoration and Reconstruction and School of Material Science and Engineering, South China University of Technology, Guangzhou, 510640 P. R. China; 3 Department of Chemistry & Biochemistry, Stephenson Life Sciences Research Center, University of Oklahoma, 101 Stephenson Parkway, Norman, OK 73019-5300 United States; 4 Guangdong Provincial Key Laboratory of Sensor Technology and Biomedical Instrument, School of Biomedical Engineering, Sun Yat-sen University, Guangzhou, 510080 P. R. China; 5 Department of Chemistry, The Hong Kong University of Science and Technology, Clear Water Bay, Kowloon, Hong Kong P. R. China; 6 Guangzhou Regenerative Medicine and Health Guangdong Laboratory, Guangzhou, 510005 P. R. China; 7 School of Materials Science and Engineering, Zhejiang University, Hangzhou, Zhejiang 310027 P. R. China

**Keywords:** Biomedical materials, Sexual dysfunction, Biomaterials - cells

## Abstract

Injury of corpus cavernosa results in erectile dysfunction, but its treatment has been very difficult. Here we construct heparin-coated 3D-printed hydrogel scaffolds seeded with hypoxia inducible factor-1α (HIF-1α)-mutated muscle-derived stem cells (MDSCs) to develop bioengineered vascularized corpora. HIF-1α-mutated MDSCs significantly secrete various angiogenic factors in MDSCs regardless of hypoxia or normoxia. The biodegradable scaffolds, along with MDSCs, are implanted into corpus cavernosa defects in a rabbit model to show good histocompatibility with no immunological rejection, support vascularized tissue ingrowth, and promote neovascularisation to repair the defects. Evaluation of morphology, intracavernosal pressure, elasticity and shrinkage of repaired cavernous tissue prove that the bioengineered corpora scaffolds repair the defects and recover penile erectile and ejaculation function successfully. The function recovery restores the reproductive capability of the injured male rabbits. Our work demonstrates that the 3D-printed hydrogels with angiogenic cells hold great promise for penile reconstruction to restore reproductive capability of males.

## Abstract (web summary)

Injury of corpus cavernosa results in erectile dysfunction, and repair leading to restoration of function is difficult. Here the authors construct 3D printed hydrogel constructs seeded with HIF-1α-expressing muscle derived stem cells to restore corpus function in a rabbit model.

## Introduction

The cavernous tissue is an important component of the penis. A healthy cavernous tissue with an intact cavernous sinusoid structure is a prerequisite for maintaining normal erectile function and urinary function of the penis^1^. As a terminal organ, the corpus cavernosum cannot be repaired via regeneration. The repair of injured corpora cavernosa has always been difficult due to a variety of anatomical, aesthetic, and functional challenges^2^. Allopenis transplantation, a reported approach, is controversial because of ethical issues^3,4^. Acellular matrix replacement also has defects because of limited sources, potential immunogenicity, and low porosity and fails to restore penile function^5^. Therefore, there is an urgent need to explore the effective regenerative therapies for cavernous reconstruction.

However, as the most desirable method for cavernous repair, the construction of bioengineered corpora cavernosa still needs to overcome some difficulties. Due to the complex structure of the cavernous sinus and the special mechanical properties necessary to meet its physiological functions, the biomaterial used for cavernous repair must possess good processability, appropriate mechanical properties, and biocompatibility which can support the adhesion, migration, and massive proliferation of cells^6^. Based on these needs, we chose to use three-dimensional (3D) printing technology, a promising tool to create 3D biomaterials with complex biomimetic structures^7–11^, and a printable bio-ink to obtain 3D-printed hydrogel scaffolds with porous structures similar to those of penile cavernous tissue. The hydrogel scaffold was selected because it could be developed to have a mechanically strong porous structure that provided a good microenvironment and space to support cell growth, adhesion and migration^12,13^, and thus structurally and mechanically match the natural corpora cavernosa. In the tissue engineering of penile corpora, the proliferated fibrous tissue, normally formed in a hypoxic or ischemic environment at the defect site, is very different from the structure and function of the original organ and can lead to a decline in organisational functions^14^. The increase of hypernomic fibrous tissue is mainly due to the insufficient oxygen supply caused by the lack and slow repair of blood vessels. In addition, the normal organisational functions of corpora cavernosa, such as erection and ejaculation, depend on the normal circulation and blood supply of the microvascular system within the cavernous sinus^15,16^. The reconstruction of the microvascular system plays a vital role in the recovery of corpora cavernosa functions^17,18^. Therefore, we have designed a biomimetic 3D-printed hydrogel scaffolds with a multi-scale porous structure, and have improved scaffolds and cells simultaneously with the goal of reducing fibrosis around the defect site and repairing the vascular-network and physiological functions of cavernous tissue.

Given that the expression of hypoxia-inducible factor-1α (HIF-1α), a key transcription activator for regulating vascular growth factors, is sensitive to normoxia^19–21^, we designed HIF-1α over-expressed muscle-derived stem cells (MDSCs) by lentiviral transfection based gene mutation. These HIF-1α mutated MDSCs not only have stable and high-level expression of HIF-1α in normoxia and hypoxia but also lead to the improved expression of angiogenesis-related factors to promote the development of neovascularization under the regulation of HIF-1α^22,23^.

**Fig. 1:**
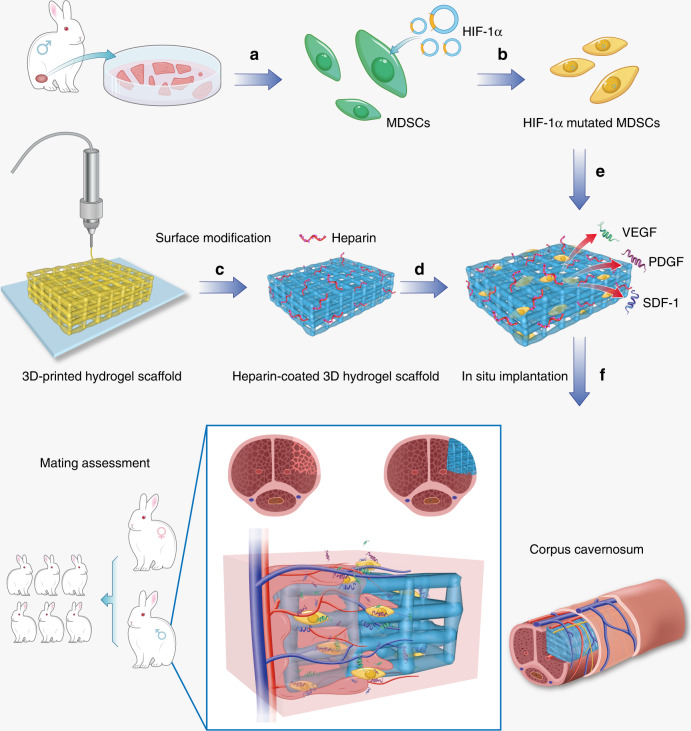
The schematic illustration of repairing injured corpora cavernosa in rabbits. **a** The MDSCs were extracted from muscles of rabbit legs. **b** The HIF-1α-mutated MDSCs were obtained by lentiviral transfection. **c** 3D-printed hydrogel scaffold was prepared by 3D printing technology. **d** Heparin was deposited onto the surface of 3D-printed hydrogel scaffold by layer-by-layer self-assembly to obtain a heparin-coated hydrogel scaffold. **e** The HIF-1α-mutated MDSCs were seeded on the heparin-coated hydrogel scaffold and secreted angiogenesis-related factors. **f** The scaffold was implanted in the rabbits with cavernosum injury to repair the penile corpora cavernosa in situ and restore penile erection and ejaculation function. Additionally, baby rabbits were born due to the recovery of the penile erection and ejaculation function (and the subsequent reproductive capability) of the male rabbits through our treatment. Our strategy can lead to the recovery of penis morphology and the reconstruction of the microvascular system in the penile corpora cavernosa.

Moreover, heparin, a negatively charged polysaccharide macromolecule with good affinity for angiogenic factors^24,25^, is known to accelerate neovascularization through binding angiogenic proteins such as vascular endothelial growth factor (VEGF) and improving their stability^26,27^. Thus we deposited heparin onto the 3D printed hydrogel scaffolds by a layer-by-layer assembly with poly-L-lysine (PLL). Then these HIF-1α mutated MDSCs were seeded onto the heparin-coated scaffolds, which were further implanted into injured corpora cavernosa. Surprisingly, angiogenesis induced by the expression of the HIF-1α and VEGF (via HIF-1α mutated MDSCs) in the hydrogel scaffolds effectively repaired the injured corpora cavernosa, and restored penile erection and ejaculation function in vivo within 4 months (Fig. 1), in sharp contrast to unsuccessful repair by the aforementioned traditional repairing methods^3–5^. Specifically, the angiogenic factors generated by HIF-1α over-expressed MDSCs were enriched on the surface of 3D-printed hydrogel scaffolds by the adsorption of heparin and then stimulated blood vessel formation in vivo. The increase in neovascularization further promote the recovery of the erection and ejaculation functions of injured corpus cavernosa. Finally, a mating assessment was conducted to verify the recovery of significant reproductive function. This study uses 3D printing technology to repair injured corpus cavernosa and may be promising to achieve effective corpus cavernosa repair in preclinical and clinical applications.

## Results

### Heparin-coated 3D-printed hydrogel scaffolds

**Fig. 2:**
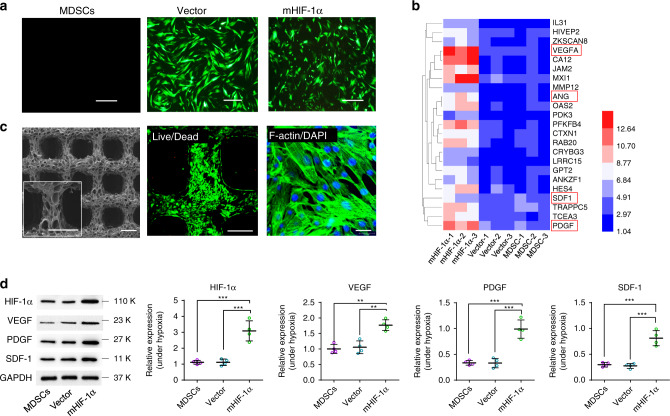
Characterization of HIF-1α gene-mutated (mHIF-1α) MDSCs. **a** Images showing three different groups of cells (MDSCs, vector and mHIF-1α groups) under fluorescence microscopy (scale bar, 100 μm) (*n* = 3). **b** High-throughput DNA sequencing reveals the difference in expression of most affected genes in cells of three different groups (MDSCs, vector and mHIF-1α groups) and is shown as a heat map (red represents highest expression level, blue represents lowest expression level) (*n* = 3). The genes in the red frames are the angiogenesis markers being studied in further experiments. **c** SEM image of 3D-printed scaffolds (left, scale bar 500 μm) as well as live/dead fluorescence image (middle, scale bar 300 μm) and DAPI/F-actin staining image (right, scale bar 30 μm) of HIF-1α gene-mutated MDSCs implanted on heparin-coated 3D-printed hydrogel scaffolds (*n* = 3). **d** Relative protein expression levels of HIF-1α, VEGF, SDF-1 and PDGF via WB detection in three different groups under hypoxia (*n* = 4). (Sidak's multiple comparisons test, two-way ANOVA. In HIF-1α, mHIF-1α vs MDSCs ***p = 0.0006, mHIF-1α vs Vector ***p = 0.0007; in VEGF, mHIF-1α vs MDSCs **p = 0.0049, mHIF-1α vs Vector **p = 0.0071; in PDGF, mHIF-1α vs MDSCs ***p = 0.0002, mHIF-1α vs Vector ***p = 0.0002; in SDF-1, mHIF-1α vs MDSCs ***p = 0.0007, mHIF-1α vs Vector ***p = 0.0005.) Data are displayed as mean ± SD and analysed by GraphPad Prism software.

A 3D-printed hydrogel scaffold was synthesised through a 3D-printing method in cooperation with ultraviolet (UV)-light crosslinking. The scaffold consisted of two biocompatible components, methacrylated hyaluronic acid (HAMA) and methacrylamide-modified gelatin (GelMA) (Supplementary Fig. 4a). The carbon–carbon double bonds were successfully grafted on hyaluronic acid and gelatin, as confirmed by ^1^H nuclear magnetic resonance (NMR) (Supplementary Figs. 1–2). The 3D-printing ink, comprising 2% HAMA, 15% GelMA, and 0.5% photoinitiator (I2959), showed good shear-thinning properties (Supplementary Fig. 3a) and an appropriate viscosity, which could be adjusted with temperature (Supplementary Fig. 3b). These properties enabled the 3D-printing ink to have good printability and be printed into hydrogel scaffolds containing a polymer network of GelMA and HAMA by the 3D printing technology (Supplementary Movie 1). The hydrogel scaffolds presented regular porous structures, as verified by scanning electron microscopy (SEM) and microscopic computed tomography (micro-CT) images (Fig. 2c and Supplementary Fig. 4b). Moreover, the compression test curves indicate that the hydrogel scaffolds exhibited prominent mechanical strength compared to that of pure GelMA, HAMA, and the traditional PEGDA hydrogel (Supplementary Fig. 4c). Repeated compression stress is the main stress to be borne by the scaffolds of cavernosa tissue. Therefore, compression test and cyclic compression test of 3D-printed hydrogel scaffolds were performed. The value of Young’s modulus of the 3D-printed hydrogel scaffolds (40.14 ± 9.03 kPa) (Supplementary Fig. 4d) is close to that of native corpus cavernosum (~20 kPa)^28^. The rigidity of polymer scaffolds, such as poly-L-lactic acid (PLA)^29^, polyglycolic acid (PGA)^30^, and their copolymer scaffolds would cause the secondary injury when the scaffolds are being compressed and rubbed with the surrounding original tissues^14^. In addition, the 3D-printed hydrogel scaffolds maintained their original mechanical properties without any damage after 20-times cyclic compression test (The stress was up to 40 kPa) (Supplementary Fig. 4e). The elevated structural stresses are in the range of 5.1–31.5 kPa during male erectile function in the dorsal aspect of the tunica albuginea^31^. Hence, these results indicate that the elevated structural stresses in penis will not damage the implanted scaffolds.

The heparin was deposited on the surface of 3D hydrogel scaffolds (Supplementary Fig. 5a) by a layer-by-layer assembly process with PLL. The success in the heparin deposition was confirmed by the zeta potential of the surface (Supplementary Fig. 5b), Fourier transform infrared (FT-IR) spectra (Supplementary Fig. 5c), and integral area of X-ray photoelectron spectroscopy (XPS) spectra (Supplementary Fig. 5d and Supplementary Table 1). In addition, both heparin-coated hydrogel scaffolds and heparin-free hydrogel scaffolds showed good biodegradability in the in vitro degradation experiments (Supplementary Fig. 5e).

The release curve of heparin (Supplementary Fig. 6b) shows that the heparin was continuously and slowly released within 30 days. The fastest rate of heparin release (0.57 μg h^−1^) was reached in the first 24 h. The amount of heparin released from our heparin-coated 3D hydrogel scaffolds (1.7–13.6 μg day^−1^ estimated from Supplementary Fig. 6b) is much less than the minimum doses of heparin (~35 units kg^−1^ or 280 μg kg^−1^) needed for reaching anticoagulant activity^32^. Therefore, the potential release of heparin from the scaffolds will not affect the clotting in animal body. To show that the heparin accumulated on the surface of the heparin-coated 3D hydrogel scaffolds is anticoagulant, the coagulation experiment was performed. The heparin-coated scaffolds have no blood clot on the surface after 30 min and the slight coagulation occurs close to edge of the wells in the heparin-coated group, while there are obvious coagulation phenomenon in heparin-free group within 4–5 min and blank group within 2 min (Supplementary Fig. 7). The results of heparin release experiment and coagulation experiment indicated that heparin-coated scaffolds have an obvious anticoagulant effect to prevent the formation of blood clots, but the anticoagulant effect is limited to the surface and vicinity of the heparin-coated scaffolds (Supplementary Figs. 6–7).

### Viability of HIF-1α gene-mutated MDSCs on scaffolds

By single-cell sequencing analysis, a heat map was generated to show the high expression of proteins in the cavernous tissue and the related gene expression pathways. HIF-1α was found to be indeed highly expressed in eight of ten cell clusters (Supplementary Figs. 8–9). We defined MDSCs transfected with the mutational HIF-1α gene (mHIF-1α) using a lentiviral vector as the mHIF-1α group (Supplementary Fig. 10a), MDSCs transfected with the empty vector as the vector group, and normal MDSCs as the MDSC group. Fluorescence imaging (Fig. 2a) of the three groups of GFP-labelled MDSCs indicated that the MDSCs were transfected successfully with a high transfection efficiency. Moreover, high-throughput DNA sequencing revealed that the HIF-1α-mutated MDSCs over-expressed more than 1000 genes than the other groups. The most affected genes related to HIF-1α, including VEGF, platelet-derived growth factor (PDGF), stromal cell-derived factor-1 (SDF-1) and angiogenin (ANG) (Fig. 2b). There were no differences in cell proliferation among the MDSCs, vector and mHIF-1α groups in the CCK8 test (Supplementary Fig. 10b), and mHIF-1α MDSCs had a lower apoptosis rate than normal MDSCs (Supplementary Fig. 11a, b). The relative protein and gene expression levels of HIF-1α related markers were analysed by quantitative real-time polymerase chain reaction (qRT-PCR) in a hypoxic environment (Supplementary Fig. 12) and by western blot (WB) tests in hypoxic and normoxic environments (Fig. 2d and Supplementary Fig. 13). The results indicated that the expression levels of VEGF, PDGF and SDF-1 in the mHIF-1α group increased significantly in comparison to that of the vector and MDSCs groups under hypoxic and normoxic conditions (Fig. 2d and Supplementary Fig. 13), consistent with the results of the gene chip (Fig. 2b) showing that the level of vascularization-related genes regulated by HIF-1α was raised. The live/dead and DAPI/F-actin staining images of cells cultured on heparin-coated and heparin-free 3D hydrogel scaffolds (Supplementary Fig. 14) and the results of the CCK8 test (Supplementary Fig. 15b) showed that heparin-coated scaffolds had good cytocompatibility. In the 3D live/dead images (Supplementary Fig. 15a) and cell migration movie (Supplementary Movie 2), the cells exhibited great viability (Supplementary Table 2) and were evenly and intensively distributed on the heparin-coated scaffolds. These results demonstrated that both heparin-coated and heparin-free 3D hydrogel scaffolds have good biocompatibility with the biological functionality to support cell adhesion, migration and proliferation. Those scaffolds are superior to the other scaffolds such as PEGDA hydrogels (only having low cytotoxicity and biosafety).

### Angiogenic capacity of cell-loaded scaffolds in mice

**Fig. 3:**
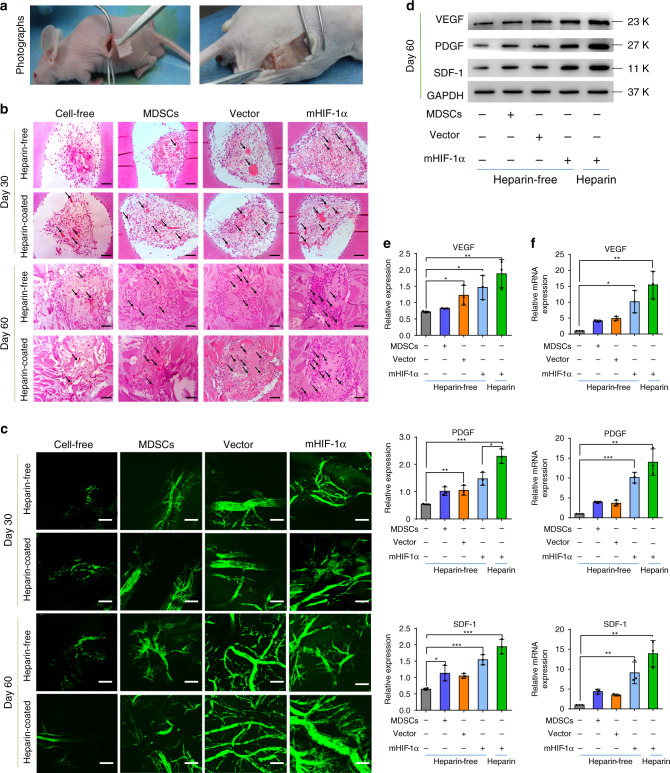
Experimental evaluation of subcutaneous implantation with cell-loaded 3D hydrogel scaffolds in nude mice. **a** Images of heparin-free and heparin-coated 3D hydrogel scaffolds loaded with different cells before and 60 days after subcutaneous implantation. **b** Images of H&E staining in the cell-free, MDSCs, vector and mHIF-1α groups after implantation in nude mice for 30 and 60 days. The scale bar is 100 μm (*n* = 3). **c** Three-dimensional angiography images of blood vessels in two implanted scaffolds after implantation in nude mice for 30 and 60 days. The scale bar is 100 μm (*n* = 3). **d**, **e** Expression of VEGF, SDF-1 and PDGF at the protein level via WB detection in the scaffold tissue after 60 days (*n* = 3). (Unpaired, two-tailed *t*-test. In **e**-VEGF, **p = 0.0093, mHIF-1α+Heparin-free vs Control *p = 0.0249, Vector+Heparin-free vs Control *p = 0.0434; in **e**-PDGF, ***p = 0.0003, **p = 0.0079, *p = 0.016; in **e**, SDF-1, mHIF-1α+Heparin vs Control ***p = 0.0006, mHIF-1α + Heparin-free vs Control ***p = 0.0006, *p = 0.0249). **f** Comparative gene expression analysis of VEGF, PDGF and SDF-1 via qRT-PCR assay in the implanted scaffold tissue after 60 days (*n* = 3). (Unpaired, two-tailed *t*-test. In **f**, VEGF, **p = 0.0045, *p = 0.0104; in **f**, PDGF, **p = 0.0025, ***p = 0.0003; in **f**, SDF-1, mHIF-1α + Heparin vs Control **p = 0.0025, mHIF-1α + Heparin-free vs Control **p = 0.0069). Data are displayed as mean ± SD. (*p < 0.05, **p < 0.01, ***p < 0.001).

These cell-loaded scaffolds and blank scaffolds were implanted in nude mice (Fig. 3a) and divided into the blank scaffolds (the cell-free group), the MDSCs-loaded scaffolds (the MDSCs group), the vector MDSCs-loaded scaffolds (the vector group), and the mHIF-1α MDSCs-loaded scaffolds (the mHIF-1α group) in heparin-coated and heparin-free scaffolds. After being harvested, the implanted scaffolds (Fig. 3a and Supplementary Fig. 16a) showed that the material and autologous tissue were completely compatible without tissue necrosis. In addition, micro-CT scanning (Supplementary Fig. 16b) showed that the scaffolds were degradable and supported tissue growth in vivo. Through haematoxylin and eosin (H&E) staining (Fig. 3b), the scaffold appeared to be completely warped with the surrounding tissue, and the internal pores were completely invaded by new tissue and blood vessels. The images of Masson’s trichrome staining show that reduced fibrotic tissue (stained with blue colour) was produced in the heparin-coated scaffolds implanted in the subcutaneous tissue of mice compared with heparin-free scaffold group (Supplementary Fig. 17). Furthermore, more new vascularized tissues were seen in the heparin-coated scaffolds than in the heparin-free scaffolds, as observed by H&E staining (Fig. 3b) and two-photon imaging, which were employed to visualize the internal new blood vessels (Fig. 3c). In particular, there were significantly more new blood vessels in the heparin-coated 3D hydrogel scaffold in the mHIF-1α group than in the other groups (Fig. 3b, c), and the compressive strength of scaffold were remained and even enhanced because the new tissue began to grow over the entire scaffold to fill the pores of the scaffold while the material was degraded (Supplementary Fig. 16b, c).

The qRT-PCR and WB tests were performed to study the molecular mechanism of the growth factors that promote vascularisation in mHIF-1α cell-loaded heparin-coated 3D hydrogel scaffolds. In WB test, the relative protein expression of angiogenesis markers (VEGF, PDGF and SDF-1) were promoted significantly in mHIF-1α MDSCs seeded on heparin-coated scaffolds than in the other groups on both day 30 and 60 (Supplementary Fig. 18 and Fig. 3d, e). The DNA expression from the qRT-PCR assay (Fig. 3f) was consistent with the results of the WB tests after 60 days. These results revealed that the heparin-coated 3D hydrogel scaffold cooperated with mHIF-1α MDSCs to exhibit good histocompatibility and favour the ingrowth of new vascular tissue (Fig. 3).

### Magnetic resonance imaging of repaired corpus cavernosa

**Fig. 4:**
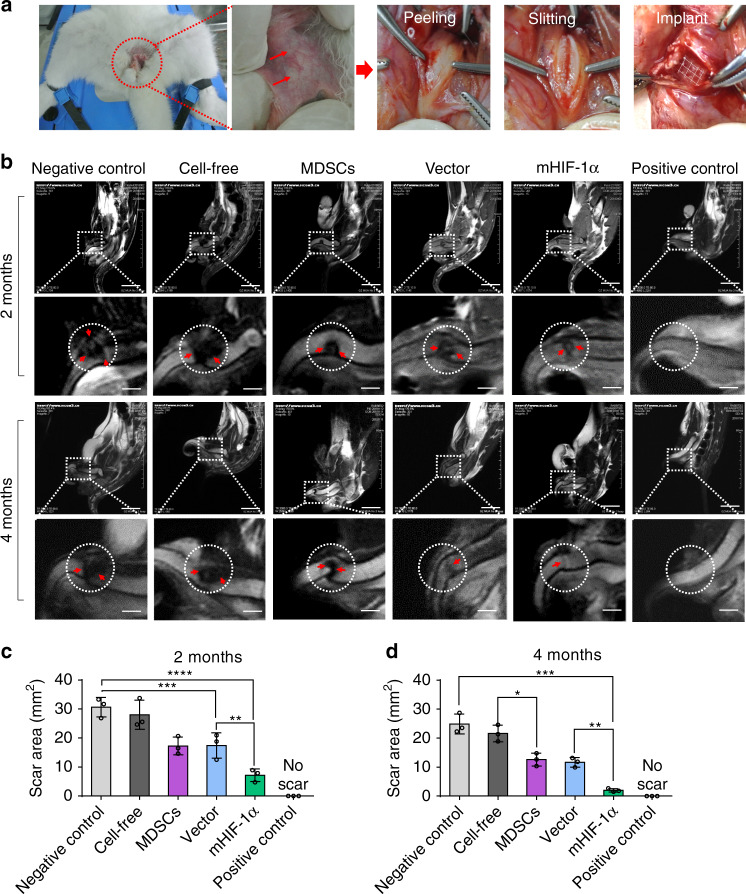
Establishment of a rabbit model of cavernosum injury and MRI examination. **a** Surgical process of implanting cell-free and cell-loaded heparin-coated 3D hydrogel scaffolds to repair injured cavernosum. **b** MRI images showing five different implantation groups (negative control, cell-free, MDSCs, vector and mHIF-1α) of the cavernosum scar area (marked as red arrows in a circle) 2 and 4 months after implantation and positive control group showing equal MRI signal. (The scale bar is 20 mm in upper images at low magnification and 5 mm in lower images at high magnification.) **c**, **d** Statistical chart of the scar area in (**b**) on month 2 (**c**) and month 4 (**d**) post implantation (*n* = 3). Data are displayed as mean ± SD and analysed by two-way ANOVA with Sidak's multiple comparisons test in (**c**) (**p = 0.0028; ***p = 0.0001; ****p < 0.0001) and **d** (*p = 0.0108; **p = 0.0055; ***p = 0.0001).

Heparin-coated scaffolds outperformed heparin-free scaffolds in angiogenesis, and thus were used to repair the defect of penile cavernosal tissue and verify whether the formation of new vessels could improve cavernous functions and accelerate erectile function recovery. The heparin-coated 3D-printed hydrogel scaffolds with designed shape (Supplementary Fig. 19, Supplementary Movie 3) were implanted to the injured site of the corpus cavernosa of a model rabbit (Fig. 4a). The implanted materials were divided into five groups, including negative control group (normal saline), cell-free group (blank hydrogel scaffolds), MDSCs group (MDSCs-loaded hydrogel scaffolds), vector group (vector MDSCs-loaded hydrogel scaffolds), and mHIF-1α group (mHIF-1α MDSCs-loaded hydrogel scaffolds). Normal male rabbits were employed as a positive control group. As expected, magnetic resonance imaging (MRI) exhibited recovery in the injured corpus cavernosa 2 and 4 months after implantation of the cell-loaded scaffolds compared to the cell-free and negative groups (Fig. 4b–d, Supplementary Table 4). Among the cell-loaded groups, mHIF-1α group showed the best performance in repairing the cavernosa defect to turn the defects into almost intact cavernosa typical for the normal group (Fig. 4b–d). At either 2 or 4 months post-operation, the mHIF-1α group had the smaller scar area than the other implantation groups (Fig. 4c, d and Supplementary Table 4). These data suggested that the mHIF-1α group promoted the healing of the injured corpus cavernosa compared to the other implantation groups.

### Histological examination of vascularization in cavernosa

**Fig. 5:**
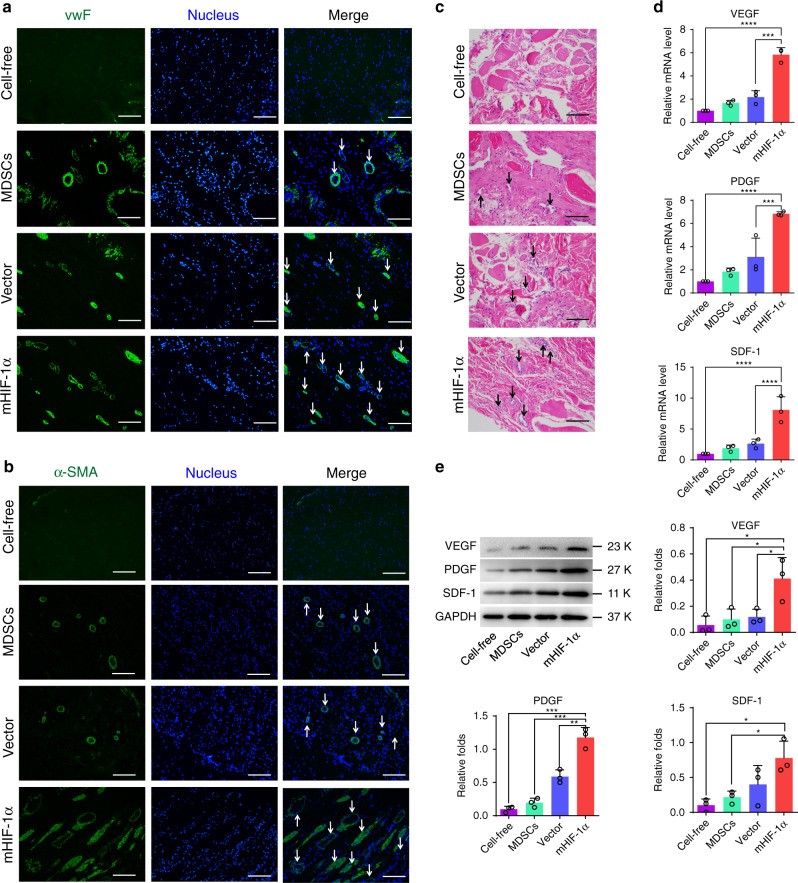
Histological and molecular biological analysis of repaired corpus cavernosa. **a** Immunofluorescence images of repaired corpus cavernosum showing that the ECs were positive for vWF (green) and cell nuclei (DAPI, blue) after 4 months of implantation (*n* = 3). (scale bar, 100 μm). **b** Immunofluorescence images of repaired corpus cavernosa showing that the SMCs were positive for α-SMA (green) and cell nuclei (DAPI, blue) after 4 months of implantation (*n* = 3). (scale bar, 100 μm). **c** H&E staining images of repaired corpus cavernosa in the cell-free, MDSCs, vector and mHIF-1α groups after 4 months of implantation (*n* = 3). (scale bar, 100 μm) **(d)** Comparative gene expression analysis of VEGF, SDF-1 and PDGF via the qRT-PCR assay for the cell-free, MDSCs, vector and mHIF-1α groups after 4 months of implantation (*n* = 3). (Sidak's multiple comparisons test, two-way ANOVA. In **d** all the ***p = 0.0001, ****p < 0.0001). **e** Protein expression of VEGF, SDF-1 and PDGF via WB detection in the cell-free, MDSCs, vector and mHIF-1α groups after 4 months of implantation (*n* = 3). (Sidak's multiple comparisons test, two-way ANOVA. In **e**, VEGF, mHIF-1α vs cell-free *p = 0.0246, mHIF-1α vs MDSCs *p = 0.0397, mHIF-1α vs Vector *p = 0.0416; In **e**, PDGF, mHIF-1α vs cell-free ***p = 0.0003, mHIF-1α vs MDSCs ***p = 0.0005, **p = 0.0050; In **e**, SDF1, mHIF-1α vs cell-free *p = 0.0104, mHIF-1α vs MDSCs *p = 0.0192). All arrows in **a**–**c** indicate the blood vessels. Data are displayed as mean ± SD.

Immunofluorescence staining for von Willebrand factor (vWF) (Fig. 5a) and alpha-smooth muscle actin (α-SMA) (Fig. 5b) and H&E staining (Fig. 5c) were used for histological examination after 4 months of implantation. The endothelial cells (ECs) and smooth muscle cells (SMCs), as the iconic cells of neovascularization, were stained positively for vWF and α-SMA, respectively. The vascular structures (indicated with arrows) were clearly more abundant in the mHIF-1α group than in the other implanted groups (Fig. 5a, b), and the result was supported by the H&E staining images (Fig. 5c). To further verify the formation of new vessels in different groups, qRT-PCR and WB tests were performed to test the expression levels of those markers related to vascularization. The expression of angiogenesis markers (VEGF, PDGF, and SDF-1) were improved in the mHIF-1α group than in the MDSCs, vector, and cell-free groups significantly (Fig. 5d, e). In addition, the expression levels of the markers related to vascularization, including HIF-1α, ANG, CD31, α-SMA, and vWF, were also risen in the mHIF-1α group compared with that of the other groups (Supplementary Figs. 21a–f and 22). This finding could be attributed to the over-expression of HIF-1α, which improved blood vessel growth into the scaffold over time.

### Erectile functions and histology of cavernosa

**Fig. 6:**
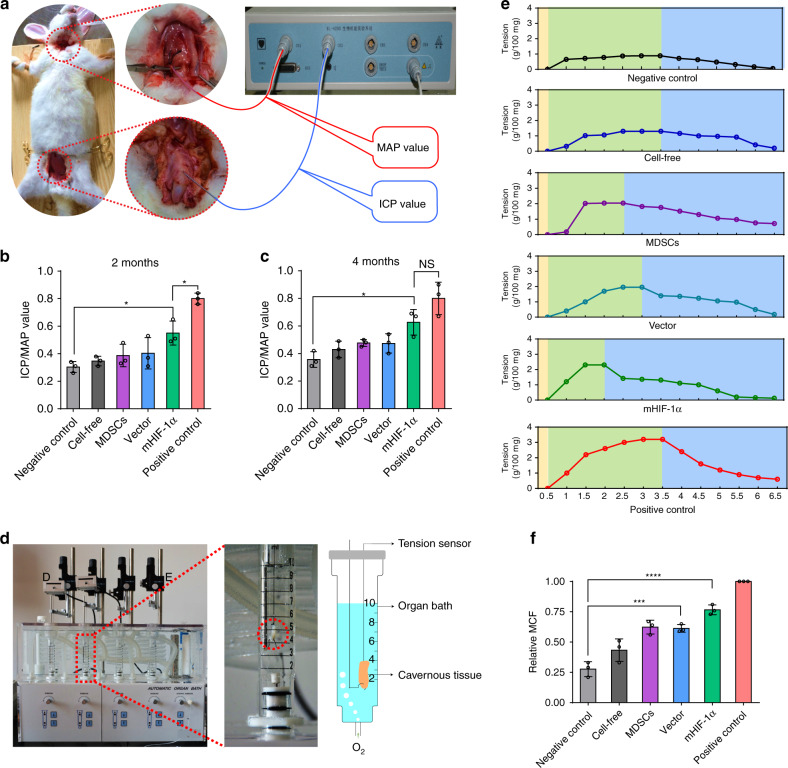
Detection of intracavernosal pressures (ICP) and mean arterial pressure (MAP) and organ bath testing in the repaired cavernosa. **a** The diagram of measurement method of ICP and MAP. **b**, **c** After 6 V of electrical stimulation was applied to the cavernous nerve, the ICP/MAP value was recorded in six different groups after 2 (**b**) and 4 (**c**) months of implantation (*n* = 3). Data are displayed as mean ± SD and analysed by two-way ANOVA with Sidak's multiple comparisons test in **b** (mHIF-1α vs Negative control: *P = 0.0482; positive control vs mHIF-1α: *P = 0.0215) and **c** (*P = 0.0276; NS, *p* > 0.05). **d** Images and schematic diagram showing organ bath testing of contraction force of repaired cavernosa at 37 °C, 95% oxygen and 5% carbon dioxide. The cavernous tissue (in the red dashed circles) was incubated in the Krebs solution and its contraction force was measured by a tension sensor under the constant oxygen flow. **e** Oscillogram of tension change by phenylephrine (green area) and phentolamine (blue area) stimulation of the repaired corpus cavernosum in each group (negative control, cell-free, MDSCs, vector and mHIF-1α or positive control group) after 4 months of implantation (*n* = 3). **f** Relative maximum contraction force (MCF) in five implantation groups (negative control, cell-free, MDSCs, vector and mHIF-1α groups) compared with that in the positive control group (mean MCF is defined as 1) after 4 months of implantation (*n* = 3). (Sidak's multiple comparisons test, two-way ANOVA, ***p = 0.0004; ****p < 0.0001). Data are displayed as mean ± SD and analysed by GraphPad Prism software.

To evaluate the erectile functions of the penile corpora cavernosa in each group, the intracavernosal pressures (ICP) and mean arterial pressure (MAP) were measured in different groups (Fig. 6a). Under an equal load of electrically stimulated cavernous nerve, the ICP/MAP values reflected the progress of erectile function. Generally, the penis in the mHIF-1α group exhibited an ICP/MAP ratio similar to that in the normal group, and the penis in the other implantation groups showed a much lower ICP/MAP ratio than the normal group (Supplementary Fig. 23, Supplementary Tables 5 and 6). Specifically, the ICP/MAP ratio of the mHIF-1α group (0.52 ± 0.06) at month 2 and (0.62 ± 0.05) at month 4 were close to that of the positive control group (0.72 ± 0.05), and the ICP/MAP values were higher than those of the other groups and much greater than those of negative control groups (Fig. 6b, c). These results suggested that the mHIF-1α group achieved a positive effect on erectile function.

Furthermore, the cavernosa of the repaired part were isolated in each group, and a contractile test was performed by drug stimulation in an automatic organ bath system on Month 4 (Fig. 6d). The maximum contraction force (MCF) and whole wave crest of the separated tissue were different in each group, clearly showing that peak systolic tension was higher in the mHIF-1α group than those of the other implanted groups and that the mHIF-1α group had analogous contractile responses compared with those of the positive control group on Month 4 (Fig. 6e and Supplementary Table 7). The mean MCF of the positive control group was defined as 1.0, and the relative MCF in the mHIF-1α group reached 0.77 ± 0.04, which was higher than that in the vector and MDSCs groups and was much better than that in the negative control and cell-free groups after 4 months of repair (Fig. 6f, Supplementary Table 8). These results indicated that the repaired cavernosa of the mHIF-1α group had optimal tissue elasticity and contraction force.

### Mating assessment

**Fig. 7:**
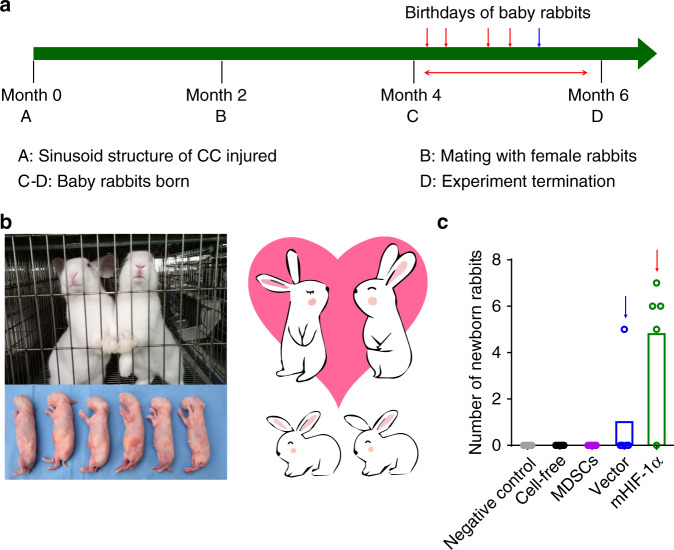
The birth time points and the numbers of newborn rabbits after different treatments in the five different groups. **a** Timeline of the experimental steps in the mating assessment. The arrows indicate the birth time points of newborn rabbits in the vector group (blue arrows) and the mHIF-1α group (red arrows). CC, corpus cavernosa. **b** The baby rabbits were born in one cage of the mHIF-1α group within after 2 months of the mating assessment. **c** The numbers of newborn rabbits in each group within 4 months of the mating assessment test (*n* = 5).

A female rabbit was mixed with each of the rabbits that had undergone the repair of cavernosa defects for two months. Four female rabbits in the mHIF-1α group gave birth to baby rabbits in succession within 4 months (Fig. 7a and Supplementary Fig. 24). No baby rabbits were born in the cell-free and negative control groups within 4 months, and only one female rabbit in the vector group gave birth to baby rabbits (Fig. 7c). The baby rabbits born in the mHIF-1α group demonstrated that the final repair effect could enable the treated male rabbits to perform normal erection and ejaculation, indicating that the function of injured cavernosa tissues in the mHIF-1α group was recovered (Fig. 7b).

## Discussion

Intact sinusoids with a regular microvascular system are the fundamental conditions of healthy cavernosa tissue with normal erection and ejaculation function^33^. Thus, the key manoeuvre of cavernosa tissue repair is to reconstruct the cavernous sinusoid-mimetic structure bearing a microvascular system and reduce. In clinical applications, traditional synthetic polymer scaffolds (e.g., poly‐L‐lactic acid (PLA)^29^ and polyglycolic acid (PGA)^30^), used as a patch material, are unable to reconstruct the cavernous functions due to their high rigidity, which would result in the secondary injury during the compression and rubbing of the scaffolds with the surrounding original tissues^14^. Up to now, there is no evidence that the polymer scaffolds have successfully repaired the erectile function of cavernosa tissue^14^. Acellular matrix-derived materials^5,34,35^ having the similar mechanical properties with the original tissues, as an advanced approach to repairing cavernous defects, are limited by their insufficient source, difficult decellularization process, and poor vascularization ability, and yield a contractile force recovery to only 63% of the normal after 6 months^5^. In our work, according to the compression test and cyclic compression tests of the 3D-printed hydrogel scaffolds, the value of Young’s modulus of the 3D-printed hydrogel scaffolds (40.14 ± 9.03 kPa) (Supplementary Fig. 4d) is close to that of native corpus cavernosum (~20 kPa)^28^. The 3D-printed hydrogel scaffolds maintain their original mechanical properties without any damage after 20 cycles of compression test (The stress was up to 40 kPa) (Supplementary Fig. 4e). And the elevated structural stresses are in the range of 5.1–31.5 kPa during male erectile function in the dorsal aspect of the tunica albuginea^31^. These results indicate that the elevated structural stresses in penis will not damage the implanted scaffolds. Our 3D-printed bioengineered scaffolds also have good biocompatibility with more than 94% survival rate of cells on them (Supplementary Fig. 14b), support the adhesion, migration, proliferation and differentiation of cells (Supplementary Fig. 14c), overcome the barrier of vascularization, and regain sufficient ICP/MAP ratio (close to that of the normal corporal tissue) for supporting copulation behaviours. Indeed, our 3D-printed hydrogel scaffolds have a multi-scale porous structure similar to the original penile cavernous tissue (Supplementary Figs. 19–20). HIF-1α is the predominant orchestrator of physiological and pathophysiological responses to hypoxia and regulates the transcription of target genes involved in angiogenesis^36^. Here, we obtain HIF-1α-over-expressing MDSCs by successfully transfecting the mHIF-1α lentiviral vector into the MDSCs. Compared to other groups loaded with normal MDSCs, the heparin-modified scaffolds that cooperated with HIF-1α-over-expressing MDSCs significantly promote the expressions of angiogenesis-relate genes and proteins (including VEGF, PDGF, and SDF-1) and the formation of neovascularisation in vivo regardless of hypoxia or normoxia (Fig. 2, Supplementary Figs. 12, 13). This distinction may be attributed to HIF-1α over-expression, which can activate more angiogenesis-related genes. In addition, the heparin on the surface of 3D-printed scaffolds can specifically bind angiogenic factors to prolong the effective time of these growth factors, then accelerate angiogenesis regeneration and increase the vascular density in tissues^25,37^. Moreover, functional evaluation and histological morphology show that HIF-1α-mutated MDSCs-loaded scaffolds could achieve a positive effect on repairing injured penile cavernosa (Figs. 4 and  5). Consequently, the ICP/MAP values of corpora cavernosa repaired by HIF-1α-mutated MDSCs-loaded scaffolds become comparable to those of  normal corpora cavernosa (Fig. 6c) and the relative maximum contraction force (MCF) is up to 77% of native corpora cavernosa (Fig. 6f). Besides, the newborn rabbits from the male rabbits treated by HIF-1α-mutated MDSCs-loaded scaffolds further confirm the recovery of physiological functions, including erectile function and ejaculation function, in the treated rabbits (Fig. 7c).

Our work represents a significant step towards developing bioengineered vascularized corpora. It is the first report on 3D-printed bioengineered scaffolds to successfully repair the defects and restore the erectile and ejaculation function, allowing the rabbits to recover their reproductive capability. Our strategy to repair cavernous tissue has outstanding value in accelerating the reconstruction of sinusoid structures. In addition to repairing the cavernosum tissue defects, these 3D-printed scaffolds have the potential to repair other vascularized tissues, such as skin, nasal tissue and myocardial tissue.

## Methods

### Preparation of heparin-coated 3D-printed hydrogel scaffolds

3D-printed hydrogels were prepared using the crosslinking of GelMA and HAMA. A mixed aqueous solution with 10% GelMA, 2% HAMA and 0.5% photoinitiator (I2959) was used as the direct bioprinting inks. The 3D-printed hydrogel porous scaffold was fabricated via a 3D-Bioplotter^TM^ (EnvisionTEC GmbH, Germany) and then exposed to ultraviolet (UV) light (3 min, 10 mW cm^−2^). PLL and heparin were coated on the surface of the 3D-printed hydrogel scaffold through the electrostatic interaction between PLL (positively charged) and heparin (negatively charged). The 3D-printed hydrogel scaffold was immersed in a 1 mg mL^−1^ PLL solution for 30 min and then rinsed twice with ultra-pure water. Next, the PLL-coated scaffold was immersed in a 10 mg mL^−1^ heparin solution for 30 min and then cleaned with ultra-pure water. The procedure was repeated 4 times to construct the heparin-coated 3D-printed hydrogel scaffold. The characterization analysis and further details of the methods are available in Supplementary Information.

### Transfection of the mHIF-1α lentiviral vector in MDSCs

The mutant human HIF-1α lentiviral vector (mHIF-1α group) (Supplementary Fig. 10a) and the empty lentiviral vector (vector group) were constructed at Hanbio company (China). MDSCs at passage 3–4 were cultured in the particular growth medium (The mixture of 10% heat-inactivated foetal bovine serum (FBS) (Gibco, Life Technologies, USA), 10% horse serum, 0.5% chicken embryo extract, and Dulbecco's modified Eagle medium (DMEM) (Gibco, Life Technologies, USA)). MDSCs were transfected with lentivirus medium at a multiplicity of infection (MOI = the quantity of lentiviruses/the quantity of cells) of 1–20, and 5 µg mL^−1^ polybrene was added to promote efficiency. MDSCs at concentrations of 30–50% were exposed to lentiviruses (vector and mHIF-1α groups) for 6–8 h, and then the lentivirus medium was replaced with normal medium. After transfected MDSCs were cultured for 48–72 h, the green fluorescent protein (GFP) expression in them, representing the success of transfection, was observed under an inverted fluorescence microscope (Fig. 2a).

### High-throughput sequencing

After the transfection of the mHIF-1α lentiviral vector in MDSCs, the pooled DNA of the HIF-1α-mutated MDSCs was sequenced by Brooks Life Sciences Company (Genewiz) using an Illumina HiSeq 2500 System with a 25-base-pair read cycle. We performed a comparative analysis of the DNA sequencing of HIF-1α-mutated MDSCs with that of the control groups (vector and MDSCs groups) (*n* = 3).

### Evaluation of the biocompatibility of scaffolds in vitro

The heparin-free and heparin-coated hydrogel scaffolds were placed in a 24-well cell culture plate and then sterilized by irradiation at an intensity of 12 kGy. Normal MDSCs, MDSCs in the vector group and MDSCs in the mHIF-1α group were seeded on these hydrogel scaffolds at a density of 10^4^ cells mL^−1^, and growth medium (1 mL) was added to each well for 7 days at 37 °C. Then, the CCK-8 assay, qRT-PCR, and WB test were performed to evaluate cell proliferation and differentiation behaviour. Later, those scaffolds seeded with these three different cells (MDSCs, vector and mHIF-1α groups) were implanted into nude mice. The qRT-PCR and WB tests were performed to investigate the angiogenesis-relevant gene expression of the MDSCs seeded on heparin-free and heparin-coated 3D-printed scaffolds. More details of the operational approaches are available in Supplementary Information.

### Establishment of a cavernous injury rabbit model

All animal experiments were approved by the Animal Ethical and Welfare Committee of South China University of Technology and followed the guidelines of National Institutes of Health. Eighty 6-month-old New Zealand male rabbits were used to generate the corpus cavernosum injury model. After anaesthetization, the area around the penis was disinfected, and the penis was covered with a surgical drape. When the root of the penis was dragged, the left bottom of the skin and fascia was opened, and then a 5 mm longitudinal incision was made in the albuginea. The cantle of the cavernous tissue was removed, and a cell-loaded heparin-coated hydrogel scaffold was transplanted into the tissue gap. The penis was lightly sutured with white membrane and skin, and the relevant groups were named according to the different materials implanted.

### The ratio of ICP and MAP

To evaluate the functions of the corpus cavernosum, the ICP value of male rabbits in different groups were measured after 2 and 4 months of implantation. The male rabbits were placed in the face up position after anaesthetization. The carotid artery was calculated by a PE-50 tube connected with a 3-way stopcock to record the MAP value. Similarly, a needle (23-gauge) connected with the sensor was inserted into the corpus cavernosum to record the intracavernosal pressure. The cavernosal nerves were directly stimulated by a bipolar stainless-steel hook-shaped electrode at 6 V, 25 Hz, duration 50 s, and pulse width 2 ms. All the data were visualized by a BL420S Biological signal recording and analysing system (Techman Software Co., Ltd., Chengdu, China). 50 IU heparinized saline was added into the tube to prevent coagulation.

### Organ bath testing of contraction forces of repaired cavernosa

The systolic/diastolic performance of the repaired corpus cavernosum was explored in vitro. First, a Krebs solution (4.7 mM KCl, 119 mM NaCl, 2.5 mM CaCl_2_, 1.2 mM MgSO_4_, 1.2 mM NaH_2_PO_4_, 25 mM NaHCO_3_, and 11 mM glucose) was prepared in advance, and an automatic organ bath (ML0146/10-220, Australia) with a 10 mL container at 37 °C, 5% carbon dioxide and 95% oxygen was used, and the parameters of Labchart7 were set at 10 mV and 20 Hz. After anaesthetization, longitudinal unstretched sections of the cavernosum (5 mm × 2 mm) were placed in an organ bath tube. Each strip maintained a basal tension of 1 g for 1 h, the basal tension value was adjusted to 0, and then phenylephrine (10^−5^ mM) was added to observe the systolic pressure. After the plateau stage for 30 s was reached, phentolamine (10^−5^ mM) was added to an organ bath tube to observe the diastolic pressure. The change in contraction was valued as 1 g force per 100 mg of corpus cavernosum (g/100 mg).

### Mating assessment

Each group of randomly repaired rabbits was paired with one female 2 months after the surgery and housed together for 4 months. No additional mating was attempted, and original feeding conditions were maintained. A rabbit birth indicated successful mating and signalled practical therapeutic effects.

### Statistical analysis

All the data were repeated at least three times. The statistics were presented as the mean ± standard deviation (SD) (*n* ≥ 3) and analysed by the GraphPad Prism 6 software.

### Reporting summary

Further information on research design is available in the Nature Research Reporting Summary linked to this article.

## Supplementary information

Supplementary Information

Description of Additional Supplementary Files

Supplementary Movie 1

Supplementary Movie 2

Supplementary Movie 3

Reporting Summary

## Data Availability

All data supporting the findings of this study and their Supplementary Information files are reported in this paper and also available from the authors upon request.
