# Supplementary material for: Functional reconstruction of injured corpus cavernosa using 3D-printed hydrogel scaffolds seeded with HIF-1α-expressing stem cells
Source: Nat Commun. 2020 Jun 1;11:2687. doi: 10.1038/s41467-020-16192-x (PMC7264263; doi:10.1038/s41467-020-16192-x)
Supplement: Supplementary file 1 — Supplementary Information [file 41467_2020_16192_MOESM1_ESM.pdf]

Supplementary Information

Functional reconstruction of injured corpus cavernosa using 3D-printed hydrogel scaffolds seeded with HIF-1α-expressing stem cells

Geng An *et al.*

Supplementary Methods

Preparation of gelatine methacryloyl (GelMA) and methacrylated hyaluronan (HAMA). GelMA was prepared as previously reported.[1,2] In short, 3 g of gelatine (~300 g Bloom, from porcine skin, Sigma-Aldrich, USA) was dissolved in 120 mL of DPBS solution at 50 °C. Then, methacrylic anhydride (6 mL) was added dropwise, and the acylation reaction occurred under continuous stirring for 3 h at 50 °C. The reaction was terminated by dilution with the addition of 200 mL of warm DPBS (40 °C). Then, the mixture was dialysed for ~7 days against deionised water to remove unreacted reagents and by-products (12 ~14 kDa cut-off dialysis membrane, 40 °C). Finally, GelMA was obtained after lyophilisation and stored at -20 °C. The preparation process of HAMA is similar to that of GelMA.[3] Four grams of hyaluronate sodium were dissolved in 300 mL of deionised water and stirred at 25 °C until fully dissolved. Then, methacrylic anhydride (10 mL) was added dropwise at 25 °C and the pH of the solution was adjusted to 8.0-9.0 with a 5 M NaOH solution. Afterwards, the reaction mixtures were stirred overnight at room temperature. The crude products were first collected by crystallising and precipitating with cold ethanol. Then, the products were re-dissolved in a small amount of deionised water and dialysed against deionised water for 7 days (12~14 kDa cut-off dialysis membrane, 25 °C). Finally, a sponge-like white solid was obtained after lyophilisation (-80 °C, 2 days), which is HAMA. The synthetic GelMA and HAMA were dissolved in D_2O (10 mg mL^{-1}) and characterised by ^1H NMR (400 MHz, Bruker, Germany) (Supplementary Figs. 1-2).

3D printing of hydrogel scaffolds. The direct printing inks were prepared by dissolving GelMA, HAMA and a photoinitiator (I2959) in deionised water or another solution (e.g., DMEM) according to the mass concentrations (w/v) of 10%, 2% and 0.5%, respectively, and the bubbles were removed after mixing. To evaluate the rheological behaviour of the 3D-printed hydrogel (consisting of 10% GelMA and 2% HAMA) printing inks, the viscosity parameters of the 3D-printed ink respond to temperature (from 5 °C to 37 °C) were recorded by rotational viscometry with plate diameter 50 mm, distance 0.108 mm at 5 rad s^{-1} (Supplementary Figure 3a). The viscosity dependence of different shear rates was investigated at 28 °C between 0.1-100 s^{-1} (Supplementary Figure 3b). The ink could be printed directly via a 3D-Bioplotter™ (EnvisionTEC GmbH, Germany). The multi-component crosslinked 3D-printed hydrogel scaffold consisting of

HAMA and GelMA was fabricated as follows. First of all, the natural cavernous sinus has a dense honeycomb-like porous structure.[4] Here, to simulate the overall shape and the internal honeycomb-like porous bionic structure of the corpora cavernosa tissue, we used computer-based 3D modeling software to design a 3D model (Supplementary Figure 21b). The designed model was stratified by 3D-Bioplotter^{TM} software, and the printing model was imported to the control interface of the 3D-printing computer software. Then, loaded the 3D-printing ink into an extrusion unit using a single-channel needle (22G, 300 μm) after the nitrogen control system was turned on. The temperature of the ink was controlled by a low-temperature control device (LTV-Dispense Head) on the 3D-Bioplotter^{TM} (EnvisionTEC GmbH, Germany). The temperature of the printing platform was maintained at 4 °C. Other printing parameters, including fibre spacing, UV irradiation time, extrusion pressure and X-Y plotting speed, were set to 800 μm, 3 min, 8×10^4 Pa and 28 mm s^{-1}, respectively, to create straight and full printing fibres. The 3D-printed hydrogel porous scaffold was initially cured by hydrogen bonding between GelMA molecules at low temperature (4 °C) and then exposed to UV light (3 min, 10 mW cm^{-2}) for further crosslinking. Finally, a 3D-printed hydrogel scaffold with an internal honeycomb-like porous structure was constructed (Supplementary Figure 21b and Supplementary Video 3).

Microcomputed tomography (micro-CT). The 3D-printed hydrogel scaffold was scanned by microcomputed tomography (XYV160H, Nikon, Japan) for collecting the micro-CT images (speed: 5° s^{-1}, 360°). The scanned images were processed by CT software (VGStudio Max 2.1) (Supplementary Figure 4b).

Preparation of acellular corporal collagen matrices (ACCM). ACCMs were obtained from the corpus cavernosum of the dead rabbits using our previously reported protocol.[5]

Scanning electron microscopy (SEM). The 3D hydrogel scaffold was observed using a 3D rotational stereoscopic microscope (HiroX7700) to analyse the shape of the scaffold and the pore distribution after the swelling equilibrium was reached in PBS at 37 °C. The acellular corporal collagen matrices (ACCM), lyophilised normal corporal tissue, and lyophilised 3D hydrogel scaffold were observed by scanning electron microscopy (MERLIN, Carl Zeiss AG, Germany) after being fixed on a sample table and sprayed with gold for 90 s.

Compression testing and repeated compression stress. The compression testing and cyclic

compression testing of the 3D-printed hydrogel scaffold were performed on a universal material testing machine (Intron5967, Instron, USA) equipped with 500 N load sensors. All hydrogel scaffolds were soaked in PBS solution at 37 °C to reach equilibrium before experiments. The compression rate was 1 mm min^{-1} and at least three samples were tested in each group. The cyclic compression test of 3D-printed hydrogel scaffolds was carried out in the sweep of 0-40% strain for 20 cycles.

Layer-by-layer self-assembly (LBL) of heparin-coated 3D-printed hydrogel scaffolds. Poly-L-lysine (PLL) and heparin were coated on the surface of the 3D printed hydrogel scaffold due to the electrostatic interactions between negatively charged heparin and positively charged PLL (Supplementary Figure 5a). The process is shown as follows. The 3D hydrogel scaffold was first immersed in a 1 mg mL^{-1} PLL solution for 30 min and then rinsed twice with ultra-pure water. Next, the PLL-coated scaffold was immersed in a 10 mg mL^{-1} heparin solution for 30 min and then cleaned by ultra-pure water. The procedure was repeated 4 times to construct the heparin-coated 3D-printed hydrogel scaffold with the surface structure of PLL and heparin.

Zeta potential during the LBL process. For the convenience of testing the surface potential during the LBL process, the LBL process of the 3D hydrogel scaffold was described as follows. The 3D-printing inks were coated on a rectangular glass sheet (2 cm x 1 cm) by spin coating (layer 0), and then layer 0 was exposed to UV light for crosslinking and curing before rinsed with deionised water 2 times. The zeta potential of layer 0 was measured using a solid-surface zeta potentiometer (Surpass) at pH = 7. Subsequently, after each modification of heparin and lysine (layer 1~9), the change values of each potential were recorded.

X-ray photoelectron spectroscopy (XPS). The surface composition of the 3D hydrogel scaffolds and heparin-coated 3D-printed hydrogel scaffolds were analysed by a multifunction photoelectron spectrometer (ISIS-300, Oxford instruments). The vacuum degree was 2 x 10^{-9} torr, and a mono-chromatic X-ray source of A1 Ka was used. The energy correction of the obtained XPS atlas was based on C1s (284.6 eV), and XPS PEAK41 software was used to fit the peaks of the characteristic elements (O, S and N) of heparin in the two groups of samples.

The release of heparin from the heparin-coated 3D-printed scaffolds. Due to the electrostatic interactions between toluidine blue O (TBO) and heparin, the surface density and

release period of heparin-coated 3D-printed scaffolds was characteried by TBO asasy.[6,7] The heparin-free scaffolds directly encapsulated with heparin served as a control group. A TBO solution was prepared by dissolving the 0.04 wt% TBO powder in 0.01 mol L^{-1} HCl/0.2 wt% NaCl aqueous solution. To establish a standard curve, 3 mL 0.04 wt% TBO solution was first added into 2 mL of a known concentration heparin solution (0.05 mg mL^{-1}, 0.1 mg mL^{-1}, 0.5 mg mL^{-1} and 1 mg mL^{-1}) and incubated for 4 h at 37 ºC on a 90 rpm shaker. Then, the heparin-TBO complex was spontaneously formed and precipitated in the mixture. The mixture was centrifuged at 8000 rpm for 10 min to remove the supernatant and the precipitate was carefully rinsed twice with HCl/NaCl solution (0.01 mol L^{-1} and 0.2 wt%, respectively). Finally, 5 mL ethanol/NaOH solution (80% and 0.2 wt%, respectively) was added to suspend the precipitate and the absorbance was recorded at 530 nm with a multifunctional microplate reader. There were five parallel samples in each group and the average value was used to make a standard curve (Supplementary Figure 6a). To determine the heparin release curve, the heparin-coated 3D-printed scaffolds were immersed in 20 mL PBS at 37 ºC on shaker (90 rpm) for 1, 4, 7, 10, 15, 25 and 30 days in a centrifugation tube. The 2 mL release medium at each time point was collected and 2 mL fresh PBS was added in the release system. Subsequently, the collected medium was added into 3 mL TBO solution and incubated for 4 h in a shaker (37 ºC, 90 rpm). Then, the quantitative characterization of heparin release was performed by using the same procedure as standard curve preparation. The released heparin density in the solution was calculated according to the standard curve, and the heparin release curve was plotted at last (Supplementary Figure 6b). Over five parallel samples were tested in each group.

Coagulation experiment. To further verify the anticoagulant effect of the scaffolds, the coagulation experiment was performed by soaking the heparin-coated scaffolds and the heparin-free scaffolds in fresh blood in the 37 ℃ shaker. The coagulation situations of different groups, including heparin-coated (heparin-coated scaffolds in the blood), heparin-free (heparin-free scaffolds in the blood) and blank (empty well with only blood) groups, were recorded at one-minute intervals.

***In vitro* degradation test.** The degradation solution was prepared by dissolving 1 U mL^{-1} hyaluronidase and 0.001 U mL^{-1} protease in PBS. The cleaned heparin-free and heparin-coated 3D

hydrogel scaffolds were immersed in 10 mL degradation solution at 37 °C on a shaker (120 rpm) for 0, 6, 20, 35, 50, 65, 80 and 100 days in a centrifuge tube. Recorded the weight of the scaffolds after lyophilisation at each time point.

Flow cytometry. After the MDSCs were cultured for 24 h under hypoxic conditions, then 1×10^6 cells were collected and resuspended in 500 μL buffer working solution. Furthermore, Comp-ANNEXIN V-APC and PI were added using the Comp-ANNFIXINV APC kit following the manufacturer's instructions (MultiSciences Lianke Biotech Co., Ltd., China). Quantitative analysis of the apoptosis proportion was conducted by flow cytometry (BD FACSAria™ III Cell Sorter No.648282, Becton Dickinson, USA) with the software Flowjo V10.

Seeding of MDSCs on scaffolds. After the MDSCs were successfully transfected with the mHIF-1α lentiviral vector and the empty lentiviral vector (as comparison), the MDSCs were cultured in a particular growth medium (high-glucose DMEM supplemented with 10% heat-inactivated FBS, 10% horse serum, 0.5% chicken embryo extract and 1% penicillin/streptomycin) in a 37 °C and 5% CO_2 humidified incubator. Normal MDSCs, MDSCs in the vector group and MDSCs in the mHIF-1α group were collected after proliferate into a sufficient number in cell culture flasks. The MDSCs in different groups were seeded on heparin-free and heparin-coated scaffolds at a density of 10^4 cells mL^{-1}, and added 1.2 mL medium to each well (24-well culture plate) for 7 days at 37 °C.

Live/dead staining and CCK-8 test. The different MDSCs were seeded on heparin-free and heparin-coated 3D hydrogel scaffolds with a density of 10^5 cells mL^{-1}. After culturing for 3 days, the cell-laden materials were stained with live/dead staining solution (calcein AM/propidium iodide) at 25 °C for 30 min and observed with confocal laser scanning microscope (CLSM, Leica TCS SP8, Germany). The CCK-8 kit (Dojindo Laboratories, USA) was employed to evaluate the cell proliferation activity of cells in the MDSCs, vector and mHIF-1α groups. Then, the cells were incubated in 0.5 mL of CCK-8 working solution at 37 °C for 1 h. Subsequently, the 450 nm absorbance density of 100 μL supernatant medium in a 96-well plate was recorded by a multifunctional microplate reader.

Cytoskeleton staining. The cell morphology on heparin-free and heparin-coated 3D-printed hydrogel scaffolds was observed by cytoskeleton staining. The procedure is described below. After

cultured for 24 h, the cells were fixed with 4% formaldehyde at room temperature for 15 min, and then washed twice by PBS. The Cell Navigator^{TM} F-actin labelling kit (AAT Bioquest, USA, green fluorescence) was used to label the cell skeleton for 1 h at room temperature and gently washed with PBS. Subsequently, the cell nuclei were stained blue by DAPI (Beyotime Institute of Biotechnology, China, blue fluorescence). Finally, the cell morphology was captured under a laser confocal microscope (TCS SP8, Leica, Germany).

Quantitative real-time PCR (qRT-PCR). Gene expression levels of specific marker were analysed by SYBR-green using a 7500 Real-Time PCR system (Applied Biosystems, Life Technologies, USA). The relative gene expression was quantified using the $2^{-\Delta\Delta CT}$ method. The primer sequences are shown in Supplementary Table 3.

Western blotting (WB). The proteins of samples were obtained from the supernatant after homogenised in a lysis buffer. After electrophoresis on 12% sodium dodecyl sulfate (SDS), the proteins was transferred to a polyvinylidene fluoride film (0.45 μm, Milliporarus, US), then blocked with 5% bovine serum for 1 h and incubated in the solution of primary antibodies against VEGF (1:700; Abcam), SDF-1 (1:700; Abcam), and PDGF-beta (1:700; Abcam) or with the GAPDH antibody (1:700; Abcam) overnight at 4 °C. Then the membranes were incubated with HRP-conjugated secondary antibodies (1:5000, Abcam). Dura SuperSignal Substrate (Pierce, USA) had been used to visualise the antigen-antibody complexes. The amount of protein was quantified using ImageJ software (National Institutes of Health, USA).

Subcutaneous implantation in nude mice in vivo. To evaluate the synergistic function of the cells and material to promote vascularisation during the tissue repair process, normal MDSCs, vector MDSCs and mHIF-1α MDSCs at 10^6 cells mL^{-1} were seeded on heparin-free and heparin-coated 3D scaffold materials (5 mm × 5 mm × 2 mm). Then, the cell-loaded scaffolds were cultured *in vitro* at 37°C for 7 days to fully adhere and spread on the surface of the material, and cell growth was observed using laser confocal microscopy. Seven-week-old nude mice were used to establish a subcutaneous implantation model in this study and divided into 5 groups (cell-free and heparin-free scaffolds, MDSCs-loaded heparin-free scaffolds, vector cell-loaded heparin-free scaffolds, mHIF-1α cell-loaded heparin-free scaffolds, and mHIF-1α cell-loaded heparin-coated scaffolds). All animal procedures were approved by the Animal Ethical and

Welfare Committee of South China University of Technology. The cell-loaded scaffold implants were implanted into the subcutaneous pocket on the backs of the mice. The skin was cut open in 1 cm incisions on bilateral sides of the spine, and the implant was completely buried under the mucous membrane of the skin. After 30 and 60 days of implantation, six mice in each group were sacrificed by the carbon dioxide method. The samples were obtained for detection analysis (Supplementary Figure 16a).

Micro-CT scanning. To trace the growth of new tissue and the changes in the pores of the scaffold after implantation, a micro-CT system (Scanco Medical, Bassersdorf, Switzerland) was employed to examine the samples, which included pre-implantation, after implantation in 30 days and 60 days.

Two-photon imaging of the blood vessels. Vascularity could be visualised in the materials through two-photon imaging technology. Primarily, the mice were anaesthetised with 1% pentobarbital (50 mg kg^{-1}) by intraperitoneal injection and fixed on the stage of a two-photon microscope (Leica, DM6000, German). To visualise the vasculature, 0.2 mL 1% fluorescein isothiocyanate (FITC, Sigma, Germany) saline solution was injected intravenously into the tail of mice. Then, the skin and fascia were carefully separated over the surface of the material, and a 2×2 mm^2 window was prepared before imaging. Two-photon images of blood vessels was obtained at a wavelength of 800 nm with a laser scanning system (Coherent, Santa Clara, CA, USA). Whole images of the X-Y-Z stacks (1024 × 1024 pixels, 2 mm resolution) were reached to 300 um below the scaffold surface to show the distribution and density of vasculature.

Bionic mechanics test of the scaffolds. After 30 days of implantation, the heparin-coated 3D hydrogel scaffold implants in the nude mice (7-week-old) were obtained (Supplementary Figure 16a). Then, a compression test was employed to assess the mechanical properties of the samples before and after implantation. The compression rate was 1 mm min^{-1}.

Haematoxylin and eosin (H&E) and Masson's trichrome staining. The samples (the scaffolds implanted in the subcutaneous or the repaired portions of cavernous tissues) were incised, fixed in 4% paraformaldehyde for 7 days, then dehydrated and embedded in paraffin, and prepared into 5-μm cross-sections, which were detected for H&E and Masson staining. Prepared sections were stained with H&E staining and Masson staining, respectively. At least 5 sections were

observed for each group.

Immunofluorescence staining. The sections were microwaved with citric acid buffer (pH 6.0) for 5 min and treated with 0.3% Triton and 10% normal goat serum blocking solution for 1 h at room temperature. Then, the sections were incubated with the PBS solution of anti-α-SMA (alpha-smooth muscle actin) (1:20; Abcam) and anti-von Willebrand factor (vWF) (1:10; Abcam) overnight at 4 °C and washed 3 times with PBS, followed by staining with the fluorescence secondary antibody (1:250, Abcam) for 1 h at room temperature. Then, the slices were incubated with DAPI for 10 min and observed under a microscope (Nikon, Eclipse Ti-S, Japan). The immunofluorescence results were analysed with ImageJ software (National Institutes of Health, Bethesda, USA).

Magnetic Resonance Imaging (MRI). MRI was used to assess the repairing effect on the corpus cavernosa structure at month 2 and 4. After anaesthetisation, 0.15 mL of a two-mixture preparation of papaverine (30 mg mL^{-1}, Northeast Pharm, China) and phentolamine (0.5 mg mL^{-1}, Shanghai Xudong Haipu, China) was injected to the root of the corpus cavernosum. After waiting for approximately 5 min, the penis was slowly congested. Then, the rabbits were examined by a 1.5 T MRI (Philips Medical Systems, Netherlands). The scar area of the injured corpus cavernosa was measured by the archived images and communication systems in each group.

Supplementary Figures

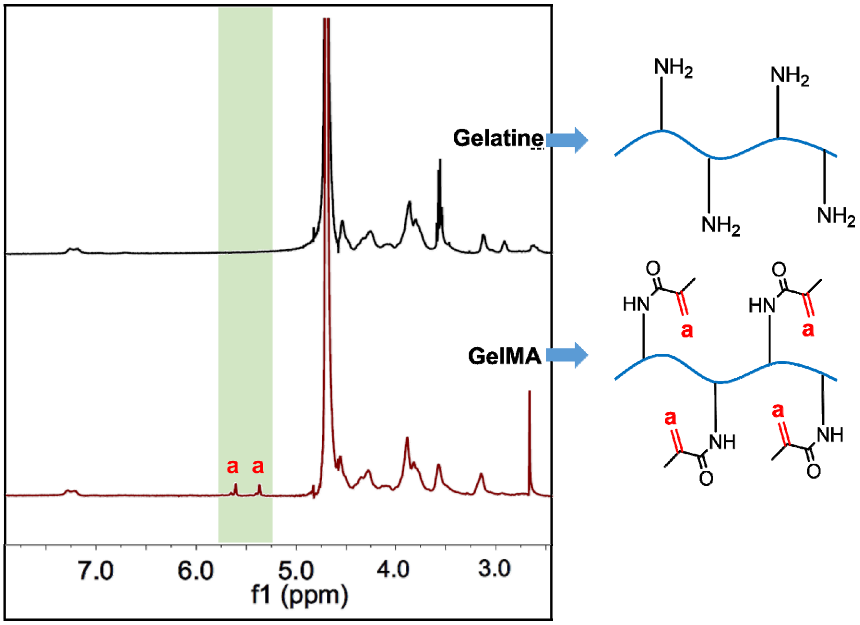

Supplementary Figure 1. ^{1}H NMR spectra (400 MHz, D_2O) of gelatine and GelMA. The peaks at 5.4-5.6 ppm correspond to the protons of the -C=CH_2 group of GelMA.

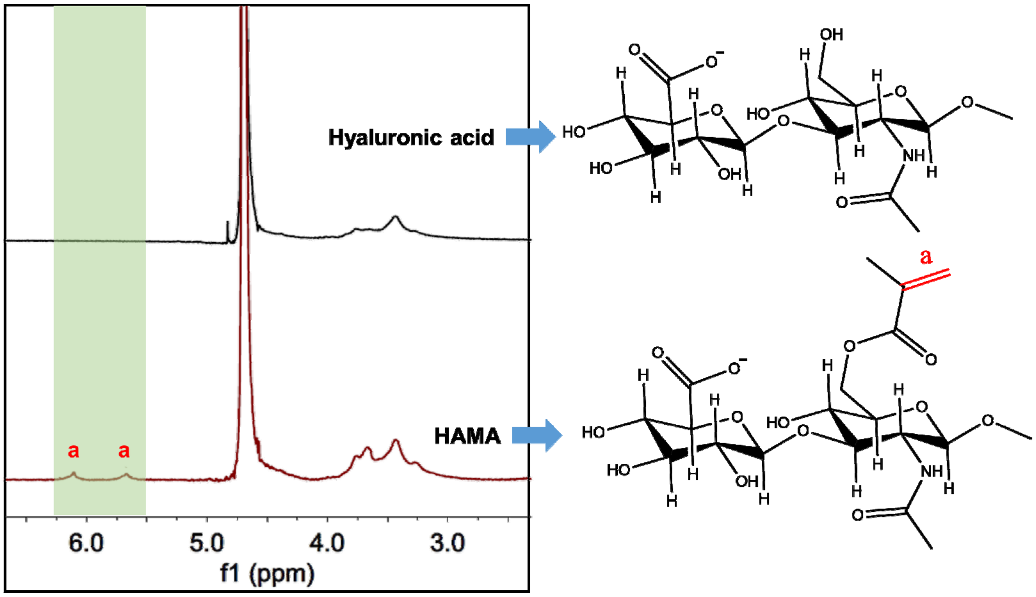

Supplementary Figure 2. ^{1}H NMR spectra (400 MHz, D_2O) of hyaluronic acid (HA) and HAMA. The peaks at 5.6–6.2 ppm represent the protons of the -C=CH_2 group of HAMA.

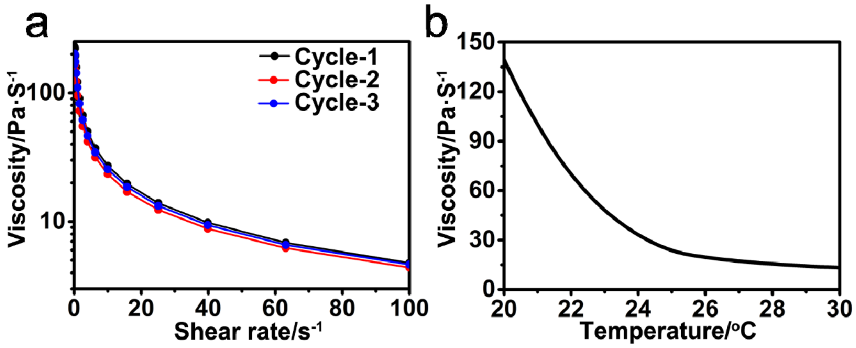

Supplementary Figure 3. Rheological properties of 3D-printing hydrogel ink. (a) Viscosity of 3D-printing hydrogel ink ranging from 10~100 Pa s^{-1} at a shear rate of 10 s^{-1}. (b) Viscosity of 3D-printing hydrogel ink with changing temperature (20 °C to 30 °C).

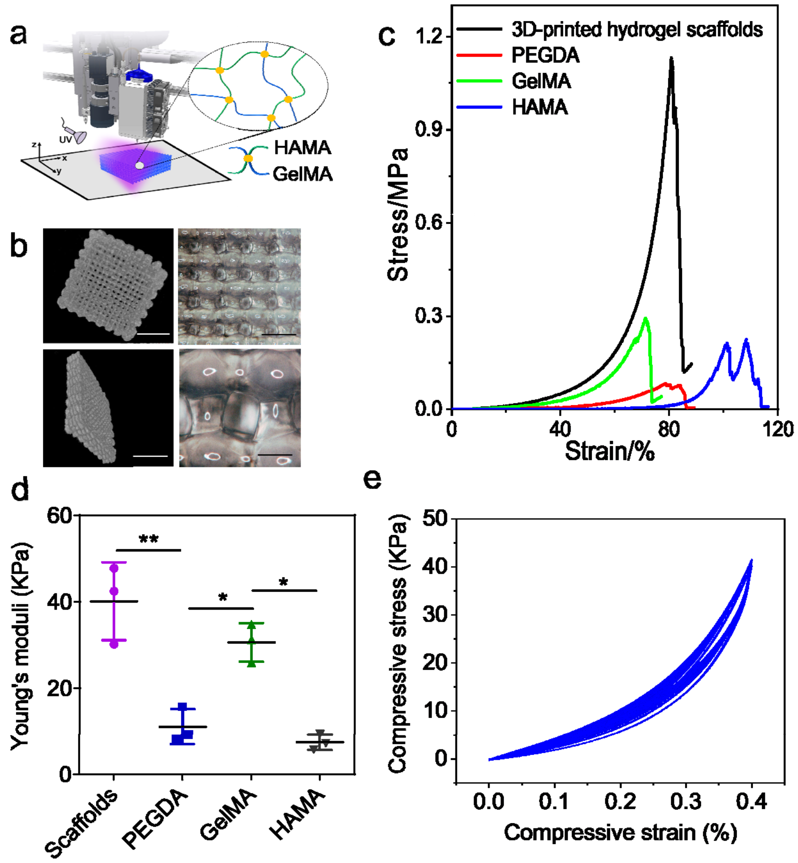

Supplementary Figure 4. Characterization of 3D-printed hydrogel scaffolds. **(a)** Schematic illustrating the 3D printing and photo-crosslinking of 3D-printed hydrogel scaffolds. **(b)** SEM images of the printed lyophilised 3D hydrogel scaffolds at different magnifications. The scale bars are 100 μm. **(c)** Stress-strain curves of various hydrogels (our 3D-printed hydrogel scaffold, PEGDA scaffolds, GelMA scaffolds and HAMA scaffolds). **(d)** Young's modulus of the 3D-printed hydrogel scaffolds (n=3). Data are displayed as mean ± SD and analyzed by the two-way ANOVA in GraphPad Prism software. (*P < 0.05, **P < 0.01). **(e)** The cyclic compression test of 3D-printed hydrogel scaffolds for 20 cycles.

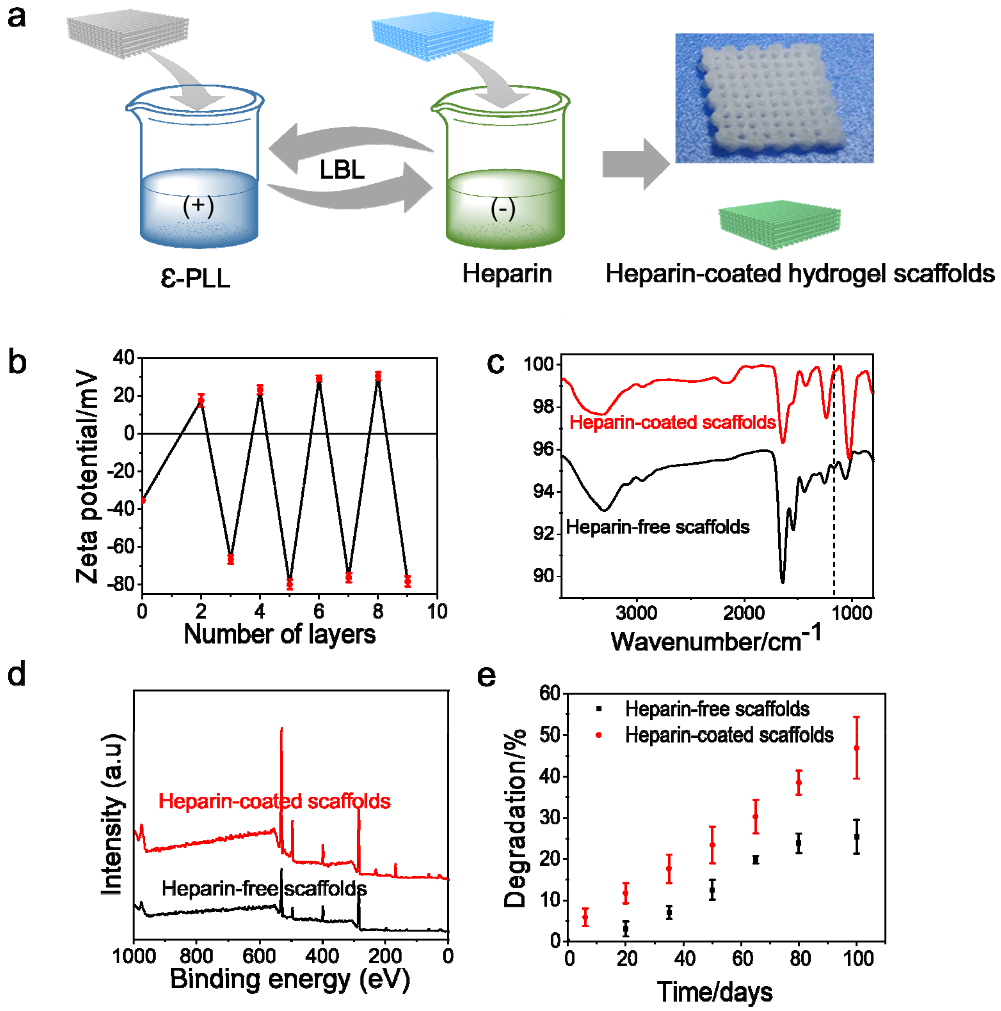

Supplementary Figure 5. Characterization of heparin-free and heparin-coated 3D hydrogel scaffolds. **(a)** Surface functionalization of a 3D hydrogel scaffold by the LBL self-assembly technology based on the electrostatic interaction between PLL and heparin. **(b)** Change in zeta potential on the scaffolds during the LBL process. **(c)** FT-IR spectrum of heparin-free and heparin-coated 3D hydrogel scaffolds. The absorption peak of C-O-S is at 1240 cm^{-1} and the peak of S=O is at 1004 cm^{-1}**. (d)** XPS spectra of heparin-free and heparin-coated 3D hydrogel scaffolds. **(e)** *In vitro* degradation kinetics curves of heparin-free and heparin-coated 3D hydrogel scaffolds (n=3). All data are displayed as mean ± SD.

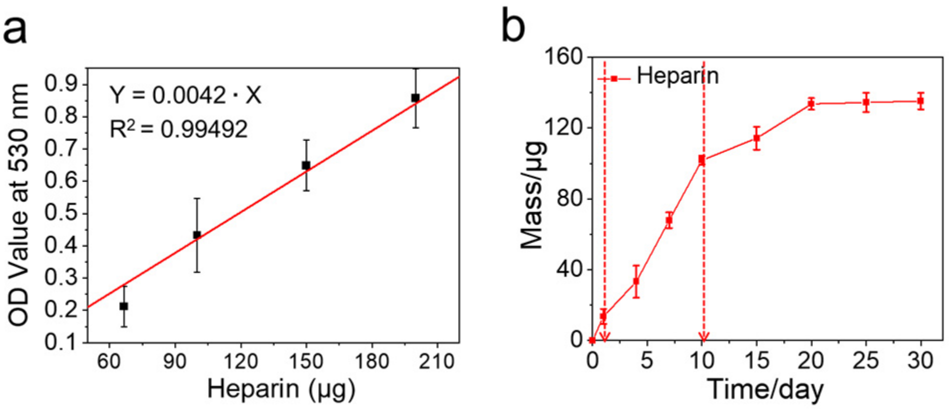

Supplementary Figure 6. The release of heparin from the heparin-coated 3D-printed scaffolds. a) The standard curve of known concentration heparin solution was characteried by TBO asasy. The absorbance of the heparin-TBO complex occurs at 530 nm (n=3). b) The heparin release curve from the heparin-coated 3D-printed scaffolds in PBS at 37 ºC (n=3). All data are displayed as mean ± SD.

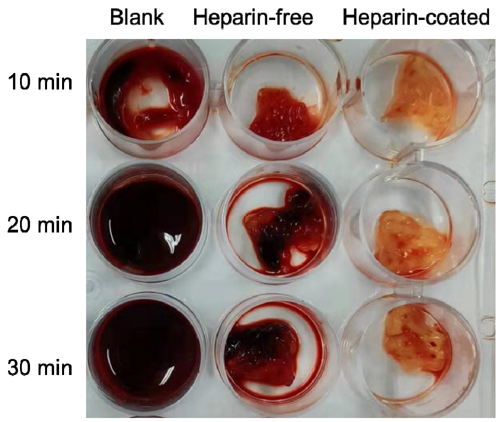

Supplementary Figure 7. The photographs of coagulation phenomenon after the heparin-coated or heparin-free scaffolds were soaked in fresh blood for 10，20，and 30 min (n=3).

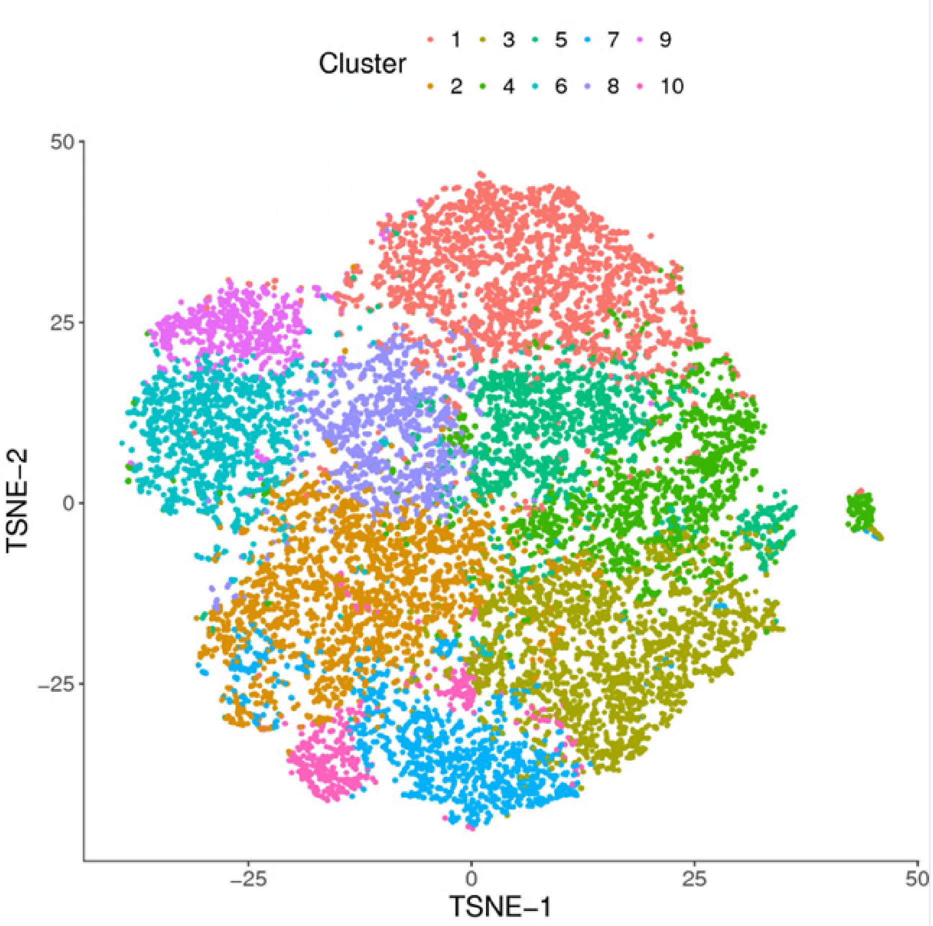

Supplementary Figure 8. The hierarchical clustering of cells in cavernous tissue was analysed by single-cell profiling.

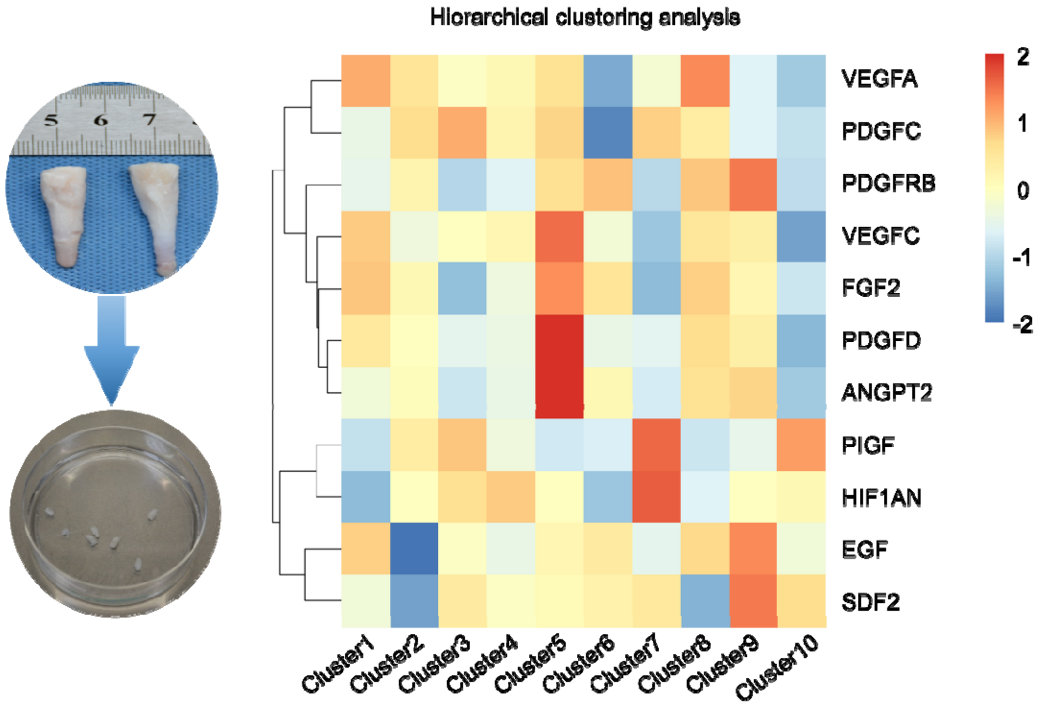

Supplementary Figure 9. Gene expression of cells in cavernous tissue was detected by single-cell profiling and is shown as a heat map (red represents highest expression level, blue represents lowest expression). Ten main clusters are shown on this dendrogram.

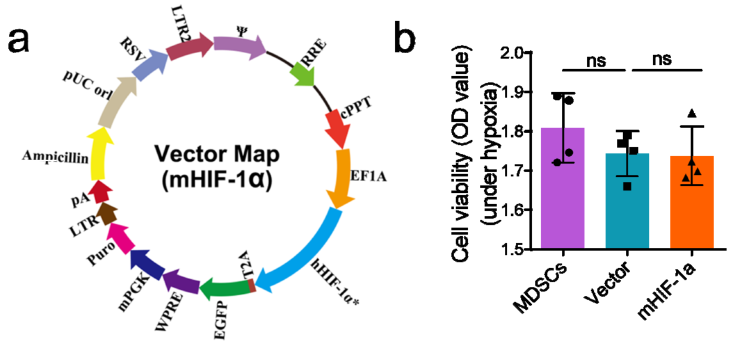

Supplementary Figure 10. Expression of HIF-1α gene-mutated (mHIF-1α) MDSCs. **(a)** Transfection of the mHIF-1α lentiviral vector in MDSCs. **(b)** OD values (450 nm) of CCK-8 assay showing the cell viability of cells in the MDSCs, vector and mHIF-1α groups under hypoxia (n=4). Data are displayed as mean ± SD and analysed by the two-way ANOVA in GraphPad Prism software. (ns, $p>0.05$)

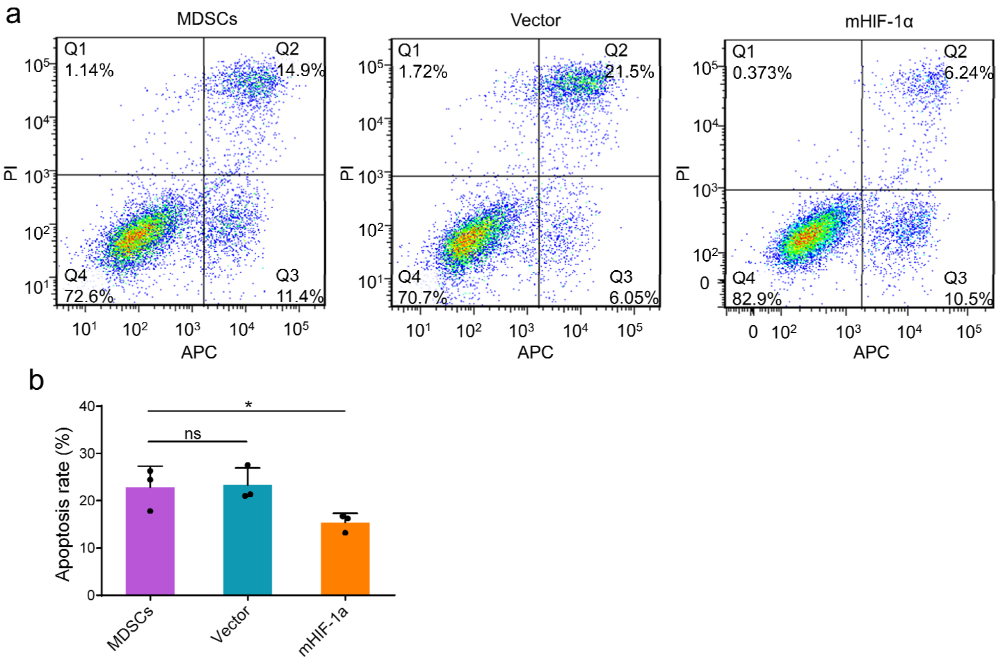

Supplementary Figure 11. Apoptosis analysis of the cells via flow cytometry. **(a)** Apoptosis level of the cells in the MDSCs, vector and mHIF-1α groups measured using flow cytometry. **(b)** Apoptosis rates of the cells in the MDSCs, vector and mHIF-1α groups according to the flow cytometry results (n=3). Data are displayed as mean ± SD and analysed by the two-way ANOVA in GraphPad Prism software. (ns, $p>0.05$; *$p < 0.05$). **(c)** WB detection of apoptosis-related proteins (BCL2 and BAX) after the cells were cultured for 24 h (n=4). Data are displayed as mean ± SD and analysed by the two-way ANOVA in GraphPad Prism software. (***$p < 0.001$, ****$p < 0.0001$).

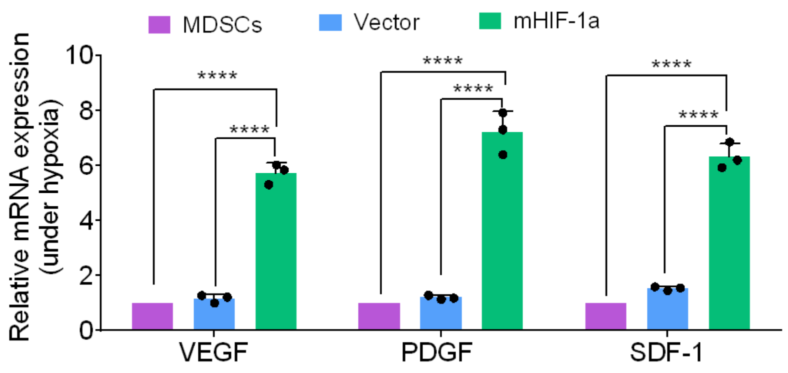

Supplementary Figure 12. Comparative gene expression analysis of VEGF, SDF-1 and PDGF via qRT-PCR assay under hypoxia (n=3). Data are displayed as mean ± SD and analysed by the two-way ANOVA in GraphPad Prism software. (****p < 0.0001).

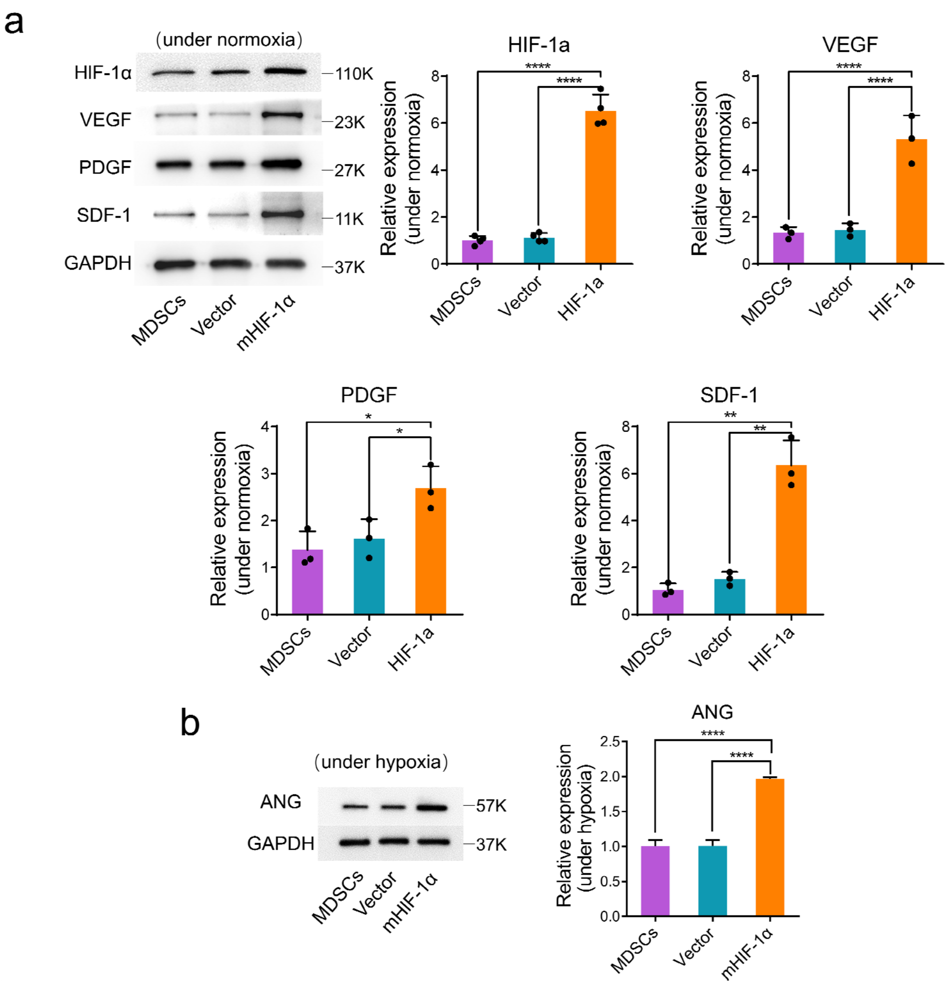

Supplementary Figure 13. Gene and protein expression of angiogenesis-related factors. (a) Relative protein expression of HIF-1α, VEGF, PDGF, and SDF-1 via WB detection under normoxia (Unpaired, two-tailed t-test, n>3; *P < 0.05, **P < 0.01, ****P < 0.0001). **(b)** Relative protein expression of ANG via WB detection under hypoxia (Two-way ANOVA, n=3). Data are displayed as mean ± SD and analyzed by GraphPad Prism software. (****P < 0.0001).

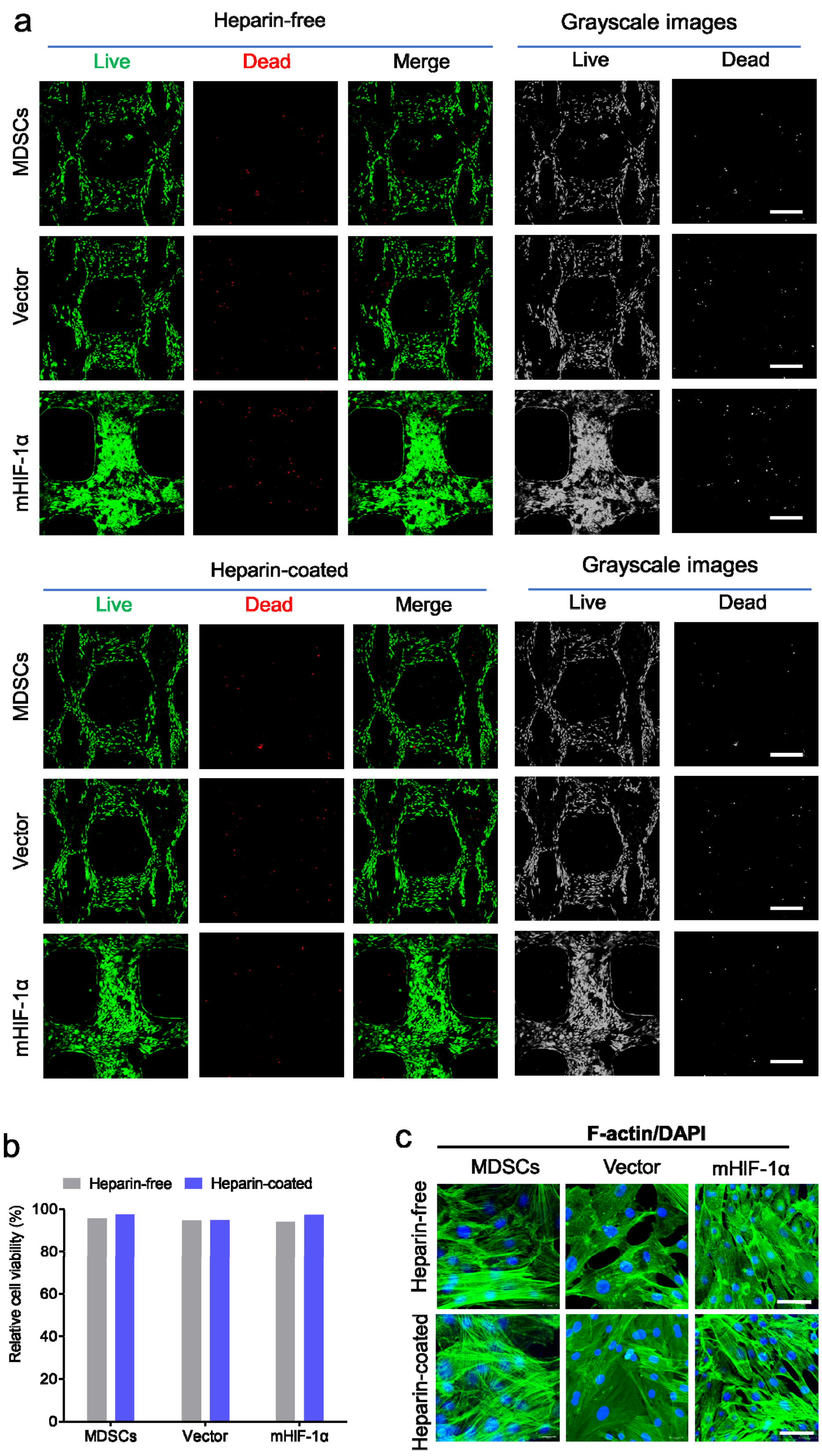
a
Heparin-free
Live
Dead
Merge
Grayscale images
Live
Dead
MDSCs
Vector
mHIF-1α
Heparin-coated
Live
Dead
Merge
Grayscale images
Live
Dead
MDSCs
Vector
mHIF-1α
b
Heparin-free
Heparin-coated
Relative cell viability (%)
100
80
60
40
20
0
MDSCs
Vector
mHIF-1α
c
F-actin/DAPI
MDSCs
Vector
mHIF-1α
Heparin-free
Heparin-coated

Supplementary Figure 14. Cell viability on the scaffolds. **(a)** Live/dead fluorescence images showing the cells in the MDSCs, vector and mHIF-1α groups after cultured on heparin-free and heparin-coated 3D hydrogel scaffolds for 3 days (scale bar, 300 μm). **(b)** Relative cell viability of the cells in the three groups after being cultured on heparin-free and heparin-coated 3D hydrogel scaffolds for 3 days. **(c)** Fluorescence images showing the morphology of cells in the MDSCs, vector and mHIF-1α groups after cultured on 3D hydrogel scaffolds for 24 h (scale bar, 60 μm) (n=3).

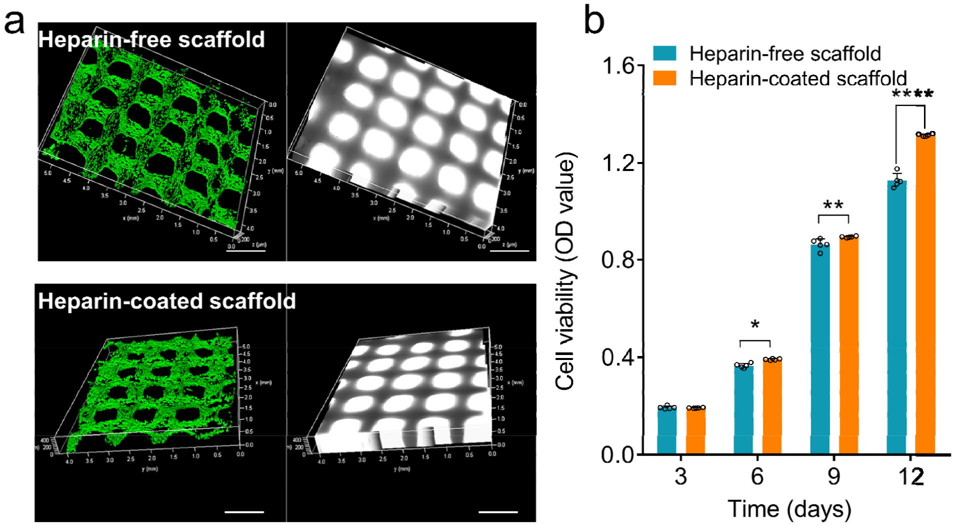

Supplementary Figure 15. Cytocompatibility of heparin-free and heparin-coated 3D hydrogel scaffolds. **(a)** Live/dead staining images of heparin-free and heparin-coated 3D hydrogel scaffolds seeded with MDSCs. **(b)** OD values (450 nm) of CCK-8 assay showing the cell viability of the MDSCs, vector and mHIF-1α groups (n=4). Data are displayed as mean ± SD and analyzed by the two-way ANOVA in GraphPad Prism software. (*P < 0.05, **P < 0.01, ****P < 0.0001).

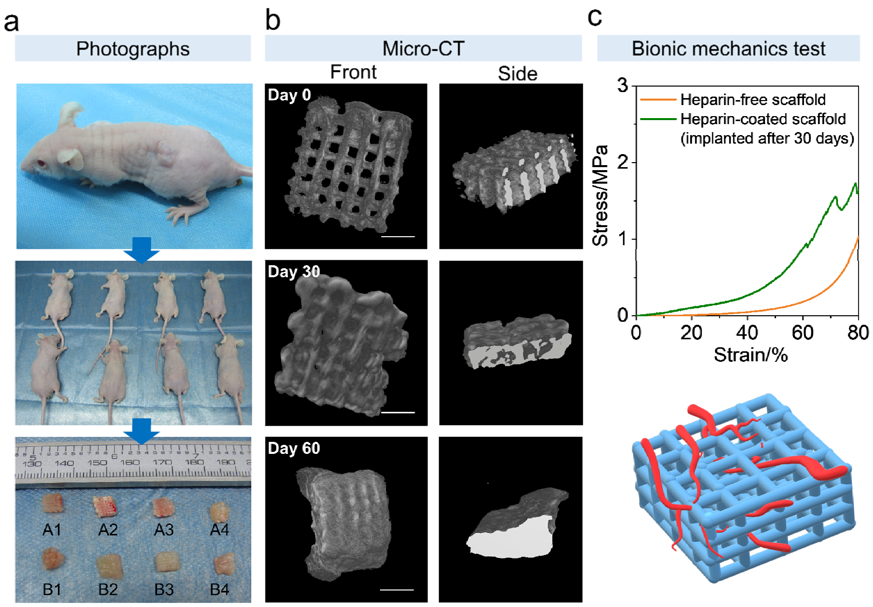

Supplementary Figure 16. Evaluation of heparin-coated 3D hydrogel scaffolds implanted in nude mice for 30 or 60 days. **(a)** Photographs of the nude mice and acquired tissue scaffolds after 30 and 60 days of implantation. **(b)** Micro-CT 3D reconstruction images of porous scaffolds after 30 and 60 days of implantation in nude mice. **(c)** Bionic mechanics test of heparin-free and heparin-coated 3D hydrogel scaffolds after 30 days of implantation in nude mice.

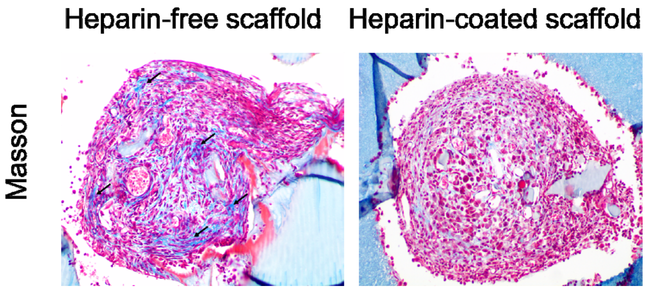

Supplementary Figure 17. The images of Masson's staining after heparin-free or heparin-coated scaffolds were implanted in the subcutaneous tissue of mice (n=3). (The fibrotic tissue was stained with blue color).

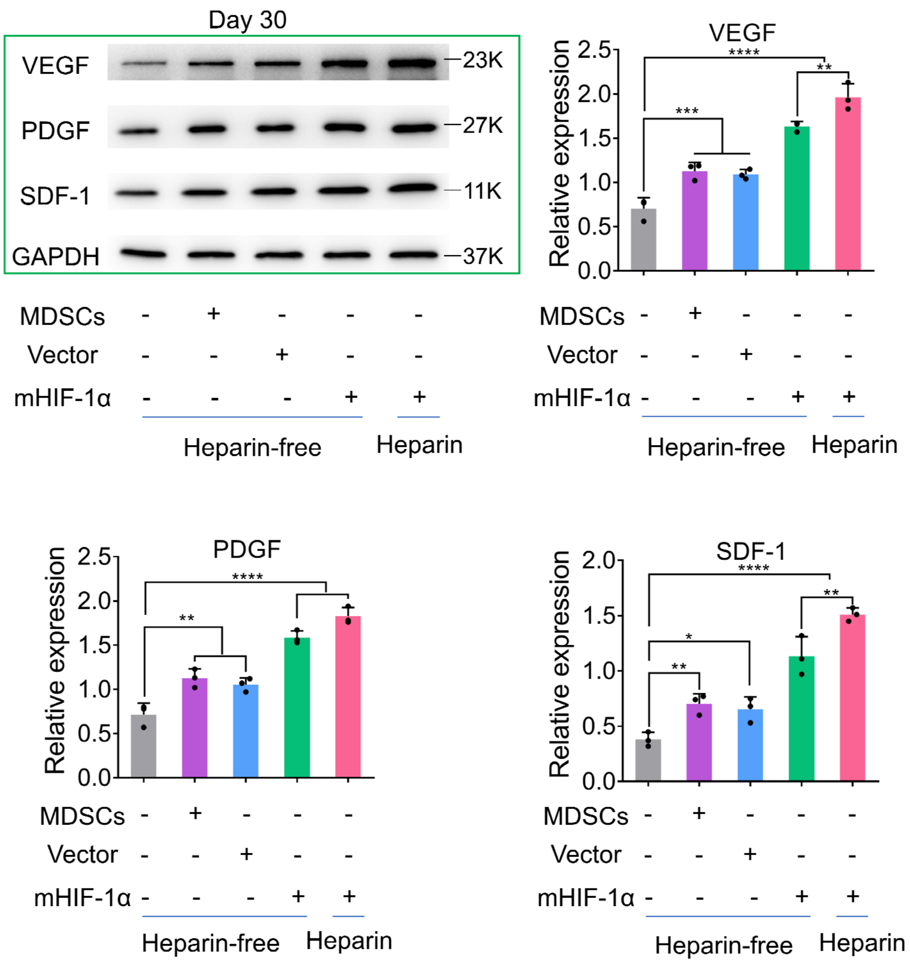

Supplementary Figure 18. The protein expression of VEGF, SDF-1 and PDGF via WB detection in the scaffold tissue after implantation in nude mice for 30 days (n=3). Data are displayed as mean ± SD and analysed by the two-way ANOVA in GraphPad Prism software. (*P < 0.05, **P < 0.01, ****P < 0.0001).

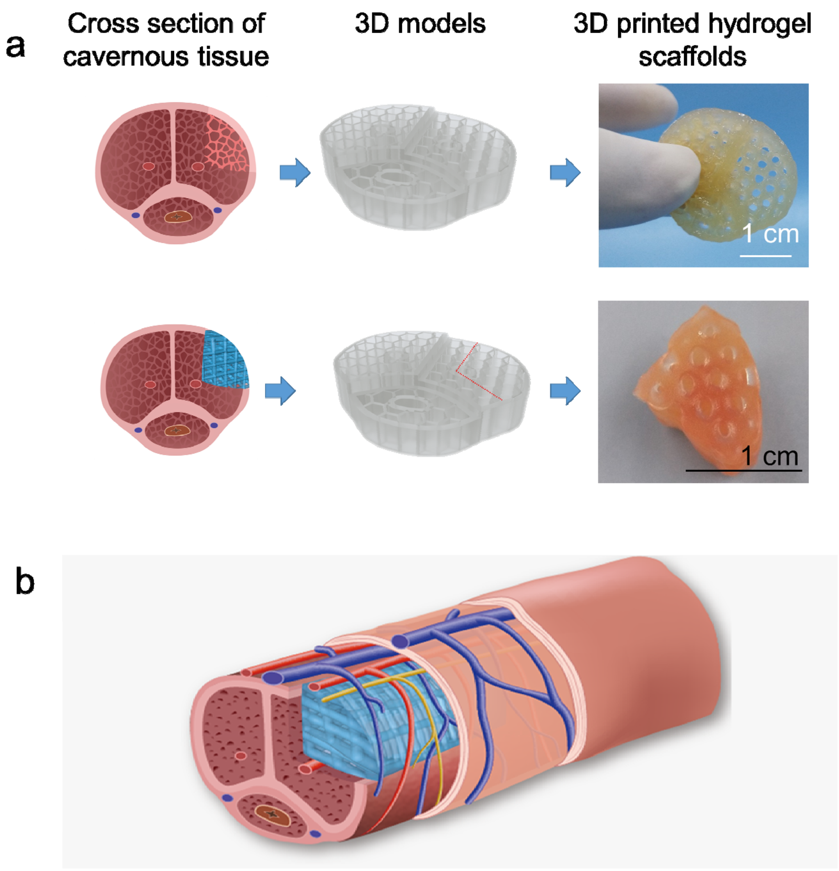

Supplementary Figure 19. The design of the cavernous sinusolid-mimetic structures of the 3D-printed hydrogel scaffolds. (a) Honeycomb porous hydrogel scaffolds simulating the cross section of cavernous tissue by the 3D printing technology. The scale bars is 1 cm. (b) Schematic illustration of 3D-printed hydrogel scaffold implanted into the injured corpora cavernosa for the repair of partial corpus cavernosa defect.

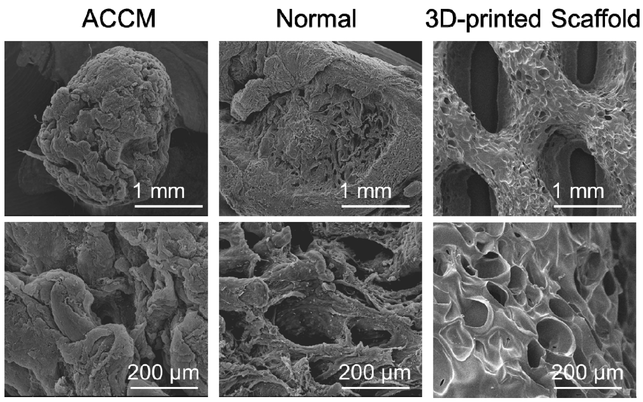

Supplementary Figure 20. SEM images of acellular corporal collagen matrices (ACCM), normal corporal tissue, and 3D hydrogel scaffold at different magnifications (n=3).

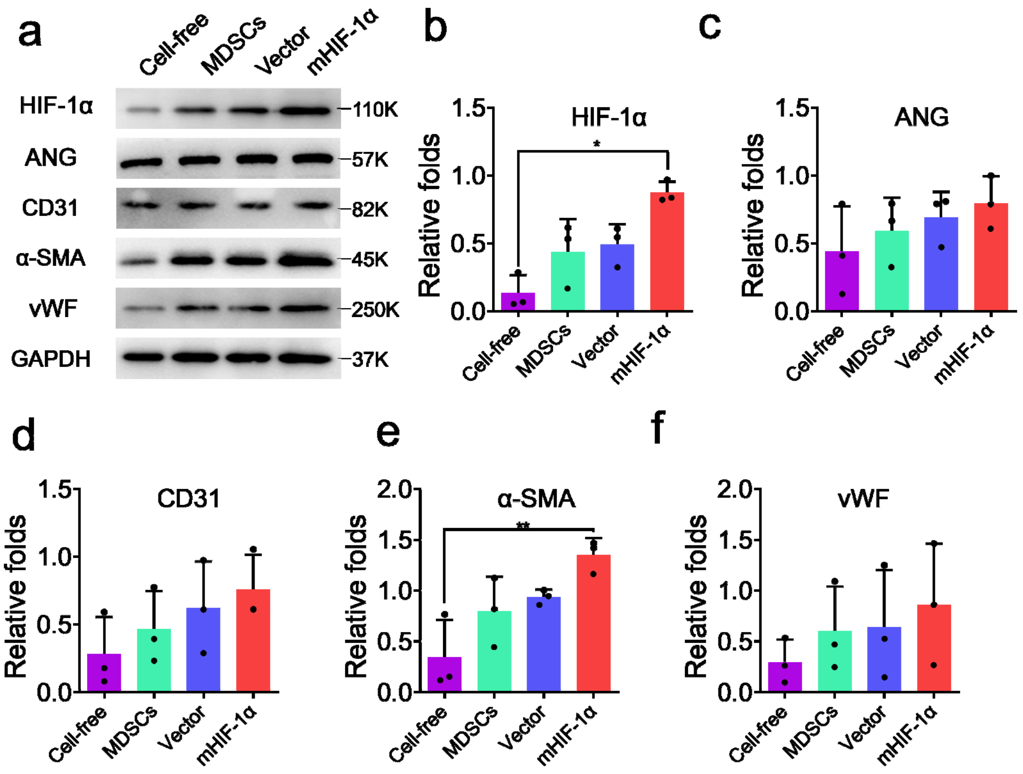

Supplementary Figure 21 Protein expression after 4 months of implantation. **a-f.** Protein expression of HIF-1α, ANG, CD31, α-SMA, and vWF via WB detection in the cell-free, MDSCs, vector and mHIF-1α groups after 4 months of implantation (n=3). Data are displayed as mean ± SD and analysed by the two-way ANOVA in GraphPad Prism software. (*P < 0.05, **P < 0.01).

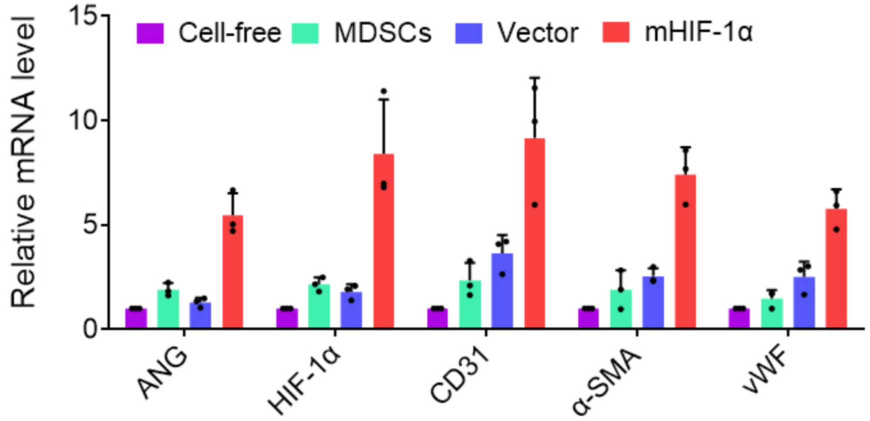

Supplementary Figure 22. Comparative gene expression analysis of ANG, HIF-1α, CD31, α-SMA, and vWF via qRT-PCR assay in the cell-free, MDSCs, vector and mHIF-1α groups after 4 months of implantation (n=3). Data are displayed as mean ± SD.

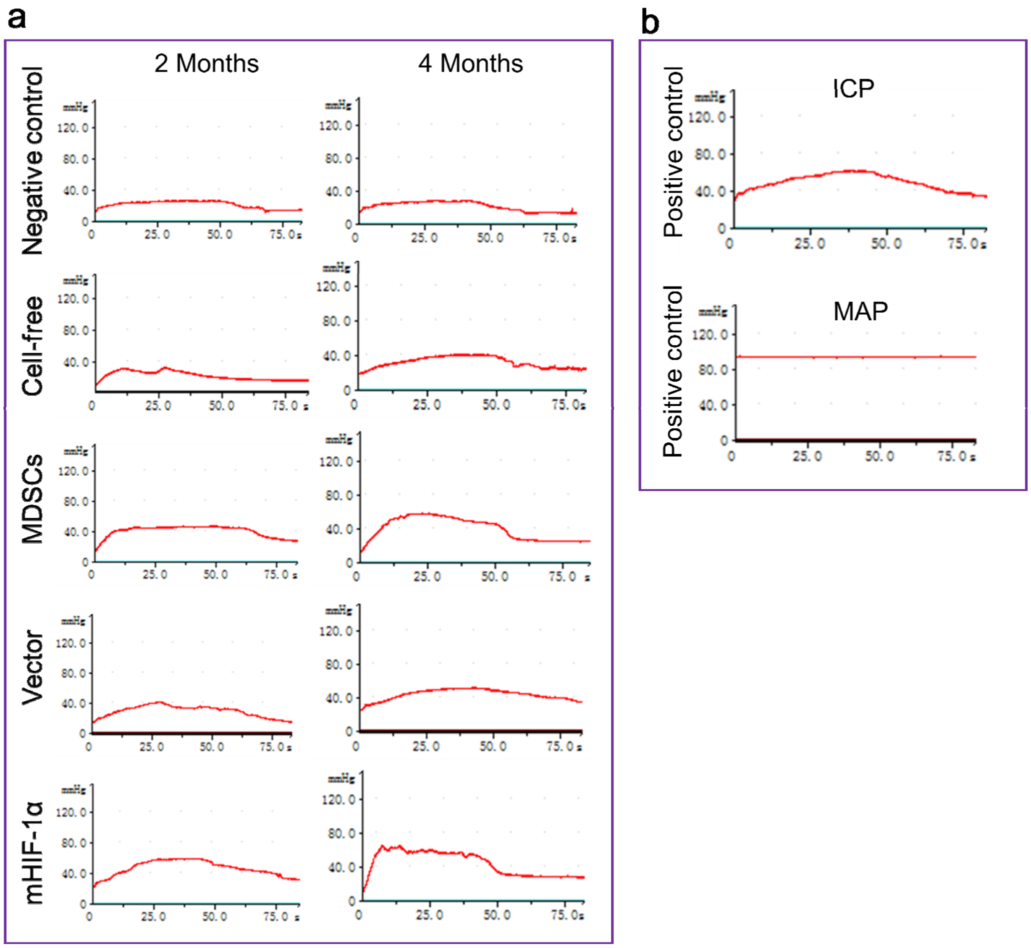

Supplementary Figure 23. Original oscillograms of ICP and MAP. **(a)** Original oscillograms of ICP in the different implanted groups after 2 and 4 months of implantation. **(b)** Original oscillogram of ICP and MAP in the positive control group.

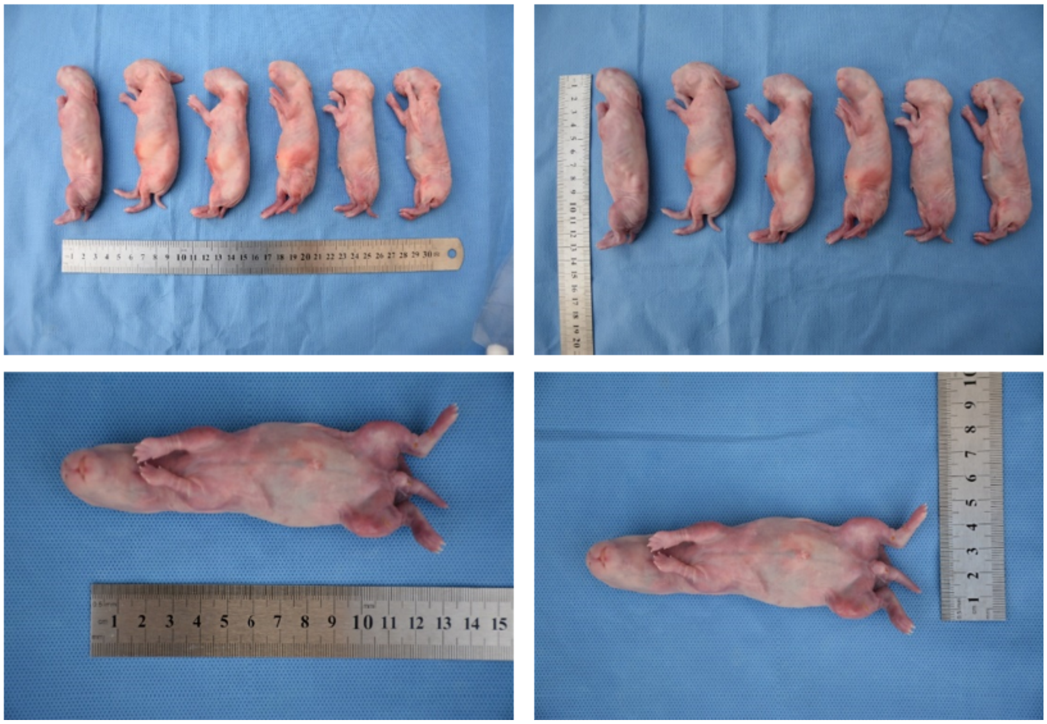

Supplementary Figure 24. Photographs of newborn baby rabbits in the mHIF-1α group after 2 months of mating experiment.

Supplementary Tables

Supplementary Table 1. Proportion of surface elements on heparin-free and heparin-coated 3D hydrogel scaffolds calculated via XPS.

Materials/%	C1s	N1s	O1s	S2p
**Heparin-free 3D hydrogel scaffolds**	51.86	8.75	36.17	0
**Heparin-coated 3D hydrogel scaffolds**	58.90	13.85	27.25	3.27

Supplementary Table 2. The quantification of the green/red (live/dead) cells in Supplementary Figure 12a.

	**Heparin-free**			**Heparin-coated**		
	Live	Dead	Survival Rate (%)	Live	Dead	Survival Rate (%)
**MDSCs**	507	23	0.96	590	16	0.97
**Vector**	594	33	0.95	508	28	0.95
**mHIF-1α**	5478	37	0.94	574	18	0.97

Supplementary Table 3. The primers used for qRT-PCR in our work.

Gene	Forward primer (5'-3')	Reverse primer (5'-3')
**HIF-1α**	TGGCAGCAACGACACAGAAA	GCAGGGTCAGCACTACTTCG
**VEGF**	CCGATCGAGACCTTGGTGGA	GCACACACTCCAGGCTTTCA
**PDGF**	CTGGACTTCCTGAGGAGCGA	GGATGATGTAGCCGCTGTCG
**SDF-1**	GCAACAACCGACAAGTGTGC	AAACAAGGCCCTGGCCAAAT
**CD31**	ATGCTCTCCAAGCCCAGGAT	TCCACGTCTTTGGTTGGGTT
**vWF**	TCCTGGTCACCGATGTCTCC	CGCTGGAGCTTCACCACATT
**ANG**	CCTCACCCTGCAAAGACACC	CCTCCCACGTGTTTGCAAGT

Supplementary Table 4. Scar area in each group on month 2 and 4 evaluated by MRI.

Time	Group	Scar area (mm^2)
**2 months**	Negative control	30.64 ± 3.36
	Cell-free	28.01 ± 5.02
	MDSCs	17.25 ± 3.08
	Vector	17.43 ± 4.38
	mHIF1-α	7.18 ± 2.17
**4 months**	Negative control	24.84 ± 3.41
	Cell-free	21.61 ± 2.85
	MDSCs	12.59 ± 2.21
	Vector	11.65 ± 1.62
	mHIF1-α	2.00 ± 0.60

Supplementary Table 5. Quantification of basic ICP and peak ICP in different groups. ICP, intracavernosal pressures.

TIME	GROUP	BASAL ICP (MMHG) (MEAN ± SD)	PEAK ICP (MMHG) (MEAN ± SD)
**2 MONTHS**	Positive control	21.33 ± 3.04	76.83 ± 5.41
	Negative control	20.20 ± 3.45	32.33 ± 4.30
	Cell-free	18.97 ± 3.62	36.67 ± 4.10
	MDSCs	18.93 ± 3.20	41.43 ± 4.61
	Vector	19.73 ± 3.33	42.97 ± 3.26
	mHIF1-α	20.83 ± 1.56	55.37 ± 5.85
**4 MONTHS**	Negative control	19.8 ± 3.94	37.98 ± 6.06
	Cell-free	19.03 ± 3.59	46.23 ± 6.40
	MDSCs	21.87 ± 1.96	50.90 ± 2.92
	Vector	20.23 ± 4.45	50.57 ± 7.09
	mHIF1-α	19.57 ± 3.81	63.80 ± 2.10

Supplementary Table 6. Ratio of ICP/MAP in the different groups. MAP, mean systemic arterial pressure.

Time	Group	ICP/MAP (Mean ± SD)
**2 months**	Positive control	0.72 ± 0.05
	Negative control	0.30 ± 0.04
	Cell-free	0.35 ± 0.04
	MDSCs	0.39 ± 0.05
	Vector	0.40 ± 0.03
	mHIF1-α	0.52 ± 0.06
**4 months**	Negative control	0.36 ± 0.06
	Cell-free	0.43 ± 0.06
	MDSCs	0.48 ± 0.03
	Vector	0.47 ± 0.07
	mHIF1-α	0.60 ± 0.02

Supplementary Table 7. Peak systolic tension of repaired cavernosa by stimulation of phenylephrine in different groups after 4 months of implantation.

Time	Group	Peak tension (g 100 mg^{-1}) (Mean ± SD)
**4 months**	Positive control	3.12 ± 0.48
	Negative control	0.86 ± 0.20
	Cell-free	1.35 ± 0.29
	MDSCs	1.95 ± 0.18
	Vector	1.92 ± 0.10
	mHIF1-α	2.40 ± 0.13

Supplementary Table 8. Relative maximum contraction force (MCF) detected by organ bath in each group compared with that of the normal control group.

Time	Group	Relative peak tension (Mean ± SD)
**4 months**	Positive control	1
	Negative control	0.28 ± 0.06
	Cell-free	0.43 ± 0.09
	MDSCs	0.62 ± 0.06
	Vector	0.61 ± 0.03
	mHIF1-α	0.77 ± 0.04

Supplementary References

1. Kolesky, D. B. *et al.* 3D bioprinting of vascularized, heterogeneous cell-laden tissue constructs. *Adv. Mater.* **26**, 3124–3130 (2014).
2. Zhifang Wang, Geng An, Ye Zhu, Xuemin Liu, Yunhua Chen, Hongkai Wu, Yingjun Wang, X. S. and C. M. 3D-printable self-healing and mechanically reinforced hydrogels with host–guest non-covalent interactions integrated into covalently linked networks. *Mater. Horizons* **1**, 96–101 (2014).
3. Eke, G., Mangir, N., Hasirci, N., MacNeil, S. & Hasirci, V. Development of a UV crosslinked biodegradable hydrogel containing adipose derived stem cells to promote vascularization for skin wounds and tissue engineering. *Biomaterials* **129**, 188–198 (2017).
4. Nakano, T. Three Dimensional Architecture of Collagen Fibrils in the Corpus cavernosum of the Crab-eating Monkey. *Okajimas Folia Anat. Jpn.* **73**, 185–194 (1996).
5. An, G., Ji, C., Wei, Z., Chen, H. & Zhang, J. Engineering of corpus cavernosum using vascular endothelial growth factor-expressing muscle-derived stem cells seeded on acellular corporal collagen matrices. *Urology* **81**, 424–431 (2013).
6. Yao, Y. *et al.* Effect of sustained heparin release from PCL/chitosan hybrid small-diameter vascular grafts on anti-thrombogenic property and endothelialization. *Acta Biomater.* **10**, 2739–2749 (2014).
7. Hinrichs, W. L. J., ten Hoppen, H. W. M., Wissink, M. J. B., Engbers, G. H. M. & Feijen, J. Design of a new type of coating for the controlled release of heparin. *J. Control. Release* **45**, 163–176 (1997).
8. de Vocht, D. E. C. M. *et al.* A systematic review on cell-seeded tissue engineering of penile corpora. *J. Tissue Eng. Regen. Med.* **12**, 687–694 (2018).
