# Supplementary material for: Functional reconstruction of injured corpus cavernosa using 3D-printed hydrogel scaffolds seeded with HIF-1α-expressing stem cells
Source: Nat Commun. 2020 Jun 1;11:2687. doi: 10.1038/s41467-020-16192-x (PMC7264263; doi:10.1038/s41467-020-16192-x)
Supplement: Supplementary file 2 — Description of Additional Supplementary Files [file 41467_2020_16192_MOESM2_ESM.pdf]

Description of Additional Supplementary Files

File name: Supplementary Movie 1

Description: 3D printing process of hydrogel scaffold

File name: Supplementary Movie 2

Description: Cell migration on heparin-coated scaffolds.

File name: Supplementary Movie 3

Description: 3D printing of the hydrogel scaffold with designed honeycomb porous structure for the repair of partial corpus cavernosa defect.
