# Supplementary material for: Functional reconstruction of injured corpus cavernosa using 3D-printed hydrogel scaffolds seeded with HIF-1α-expressing stem cells
Source: Nat Commun. 2020 Jun 1;11:2687. doi: 10.1038/s41467-020-16192-x (PMC7264263; doi:10.1038/s41467-020-16192-x)
Supplement: Supplementary file 6 — Reporting Summary [file 41467_2020_16192_MOESM6_ESM.pdf]

nature research

Corresponding author(s): Xuetao Shi and Chuanbin Mao

Last updated by author(s): Mar 27, 2020

Reporting Summary

Nature Research wishes to improve the reproducibility of the work that we publish. This form provides structure for consistency and transparency in reporting. For further information on Nature Research policies, see Authors & Referees and the Editorial Policy Checklist.

Statistics

For all statistical analyses, confirm that the following items are present in the figure legend, table legend, main text, or Methods section.

n/a	Confirmed	
☐	☒	The exact sample size (*n*) for each experimental group/condition, given as a discrete number and unit of measurement
☐	☒	A statement on whether measurements were taken from distinct samples or whether the same sample was measured repeatedly
☐	☒	The statistical test(s) used AND whether they are one- or two-sided<br>*Only common tests should be described solely by name; describe more complex techniques in the Methods section.*
☐	☒	A description of all covariates tested
☐	☒	A description of any assumptions or corrections, such as tests of normality and adjustment for multiple comparisons
☐	☒	A full description of the statistical parameters including central tendency (e.g. means) or other basic estimates (e.g. regression coefficient) AND variation (e.g. standard deviation) or associated estimates of uncertainty (e.g. confidence intervals)
☐	☒	For null hypothesis testing, the test statistic (e.g. *F*, *t*, *r*) with confidence intervals, effect sizes, degrees of freedom and *P* value noted<br>*Give P values as exact values whenever suitable.*
☒	☐	For Bayesian analysis, information on the choice of priors and Markov chain Monte Carlo settings
☒	☐	For hierarchical and complex designs, identification of the appropriate level for tests and full reporting of outcomes
☒	☐	Estimates of effect sizes (e.g. Cohen's *d*, Pearson's *r*), indicating how they were calculated

Our web collection on statistics for biologists contains articles on many of the points above.

Software and code

Policy information about availability of computer code

Data collection	The ink could be printed directly via a 3D-BioplotterTM (EnvisionTEC GmbH, Germany);The synthetic GelMA and HAMA were dissolved in D2O (10 mg/mL) and characterised by 1H NMR (400 MHz, Bruker, Germany);The 3D-printed hydrogel scaffold was scanned by microcomputed tomograph (XYV160H, Nikon, Japan);scanning electron microscopy (MERLIN, Carl Zeiss AG, Germany) and the compression and cyclic compression testing was performed on a universal material testing machine (Intron5967, Instron, USA); Ascularity could be visualised in the materials through two-photon imaging technology (Leica, DM6000, German); Organ bath testing was performed on automatic organ bath (ML0146/10-220, Australia);The scar area of the injured corpus cavernosa was examined by a 1.5 T MRI (Philips Medical Systems, Netherlands). The 3D-printed hydrogel scaffold was scanned by microcomputed tomograph (XYV160H, Nikon, Japan) and scanned images were processed by CT software (VGStudio Max 2.1) .
Data analysis	GraphPad Prism 6 was used for the generation of graphs and data analysis. Image J software (National Institutes of Health, USA) was used for the quantization of image.

For manuscripts utilizing custom algorithms or software that are central to the research but not yet described in published literature, software must be made available to editors/reviewers. We strongly encourage code deposition in a community repository (e.g. GitHub). See the Nature Research guidelines for submitting code & software for further information.

Data

Policy information about availability of data

All manuscripts must include a data availability statement. This statement should provide the following information, where applicable:
- Accession codes, unique identifiers, or web links for publicly available datasets
- A list of figures that have associated raw data
- A description of any restrictions on data availability

Data availability. All data supporting the findings of this study and their Supplementary Information files are reported in this paper and also available from the authors upon request.

Field-specific reporting

Please select the one below that is the best fit for your research. If you are not sure, read the appropriate sections before making your selection.

☒ Life sciences ☐ Behavioural & social sciences ☐ Ecological, evolutionary & environmental sciences

For a reference copy of the document with all sections, see nature.com/documents/nr-reporting-summary-flat.pdf

Life sciences study design

All studies must disclose on these points even when the disclosure is negative.

Sample size	Did a preliminary experiment first, and combined with previous experience and standards in the field, then set up the number of samples in different groups.
Data exclusions	No data exclusions were made.
Replication	All the experiments were carried out in three replicates or more.
Randomization	The animals were randomly separated into different groups.
Blinding	One person transplanted different 3D-printed materials into different animal groups while another person independently monitored and characterized the repair effects of corpus cavernosum without knowing the specimen received by each animal group.

Reporting for specific materials, systems and methods

We require information from authors about some types of materials, experimental systems and methods used in many studies. Here, indicate whether each material, system or method listed is relevant to your study. If you are not sure if a list item applies to your research, read the appropriate section before selecting a response.

Materials & experimental systems

n/a	Involved in the study	
☐	☒	Antibodies
☒	☐	Eukaryotic cell lines
☒	☐	Palaeontology
☐	☒	Animals and other organisms
☒	☐	Human research participants
☒	☐	Clinical data

Methods

n/a	Involved in the study	
☒	☐	ChIP-seq
☐	☒	Flow cytometry
☒	☐	MRI-based neuroimaging

Antibodies

Antibodies used	The following antibodies were used for WB or IF: VEGF (Abcam, ab1316, clone VG-1, lot:GR3192793-1); PDGF (Abcam, ab181341, lot: GR3188130-1); SDF-1 (abcam,ab18919, lot:GR3183126-2); HIF-1α (abcam,ab179483, clone EPR16897, lot:GR324451-1); α-SMA (abcam, ab7817, clone 1A4, lot:GR3190124-1); CD31 (abcam, ab212712, clone JC/70A, lot: GR3176841-1); ANG (abcam, ab133425, lot:GR180661-3); vwF (abcam, ab778, lot:GR3174233-2); PDGF (bioss, bs-0185R, lot:AG04053357); GAPDH (Boster, BA2913, lot:ZP9378BP78); Goat Anti-Rabbit(HRP) (Servicebio, G1213, lot: HP191602); Goat Anti-Mouse(HRP) (Servicebio G1214, lot: HP194405); Donkey Anti-Mouse(FITC) (Servicebio, GB22401, HP190834).
Validation	Each antibody was validated for the species (mouse or rabbit) and application (WB or IF) by the correspondent manufacturer. The usage was described in the Supplementary methods section of the manuscript: WB: The proteins of samples were obtained from the supernatant after homogenised in a lysis buffer, After electrophoresis on 12% sodium dodecyl sulfate (SDS), the proteins were transferred to a polyvinylidene fluoride film, then blocked with 5% bovine serum for 1 h and incubated in the solution of primary antibodies overnight at 4 °C. Then the membranes were incubated with HRP-conjugated secondary antibodies. IF: The sections were microwaved with citric acid buffer (pH 6.0) for 5 min and treated with 0.3% Triton and 10% normal goat serum blocking solution for 1 h at room temperature. Then, the sections were incubated with the PBS solution of primary antibodies overnight at 4 °C and washed 3 times with PBS, followed by staining with the fluorescence secondary antibody for 1 h at room temperature. Then, the slices were incubated with DAPI for 10 min and observed under a microscope.

Animals and other organisms

Policy information about studies involving animals; ARRIVE guidelines recommended for reporting animal research

Laboratory animals	Nude mice (7-week-old,male) were raised in standard SPF environment with ambiance, 40%-70% relative humidity and normal dark/light cycle; New Zealand rabbits (6-month-old, male) were raised in a standard clean environment.

Wild animals	This study did not involve wild animals.
Field-collected samples	This study did not involve field-collected samples.
Ethics oversight	All animal experiments were approved by the Animal Ethical and Welfare Committee of South China University of Technology.

Note that full information on the approval of the study protocol must also be provided in the manuscript.

Flow Cytometry

Plots

Confirm that:

☒ The axis labels state the marker and fluorochrome used (e.g. CD4-FITC).

☒ The axis scales are clearly visible. Include numbers along axes only for bottom left plot of group (a 'group' is an analysis of identical markers).

☒ All plots are contour plots with outliers or pseudocolor plots.

☒ A numerical value for number of cells or percentage (with statistics) is provided.

Methodology

Sample preparation	The MDSCs and transfected MDSCs (Vector and mHIF-1α) were cultured for 24 h under hypoxic condition.
Instrument	Fow cytometry (BD FACSAria™ III Cell Sorter No.648282, Becton Dickinson, USA)
Software	Flowjo V10
Cell population abundance	1×10^6 cells were collected and resuspended in 500 µLbuffer working solution
Gating strategy	The numbers on the X and Y axes are the exponents of the fluorescence of Annexin V-APC (633 nm excitation, emission collected at 660 nm) and PI (488 nm excitation, emission collected at 530 nm), respectively. Distinguish between viable cells (Both APC and PI are negative), early apoptotic cells (Positive APC PI negative), late apoptotic/necrotic cells (Both APC and PI are positive) and late necrotic cells (Negative APC and positive PI).

☒ Tick this box to confirm that a figure exemplifying the gating strategy is provided in the Supplementary Information.
